# Supplementary material for: A chromosome-level Camptotheca acuminata genome assembly provides insights into the evolutionary origin of camptothecin biosynthesis
Source: Nat Commun. 2021 Jun 10;12:3531. doi: 10.1038/s41467-021-23872-9 (PMC8192753; doi:10.1038/s41467-021-23872-9)
Supplement: Supplementary file 1 — Supplementary Information [file 41467_2021_23872_MOESM1_ESM.pdf]

A chromosome-level *Camptotheca acuminata* genome assembly provides insights into the evolutionary origin of camptothecin biosynthesis

Kang *et al.*

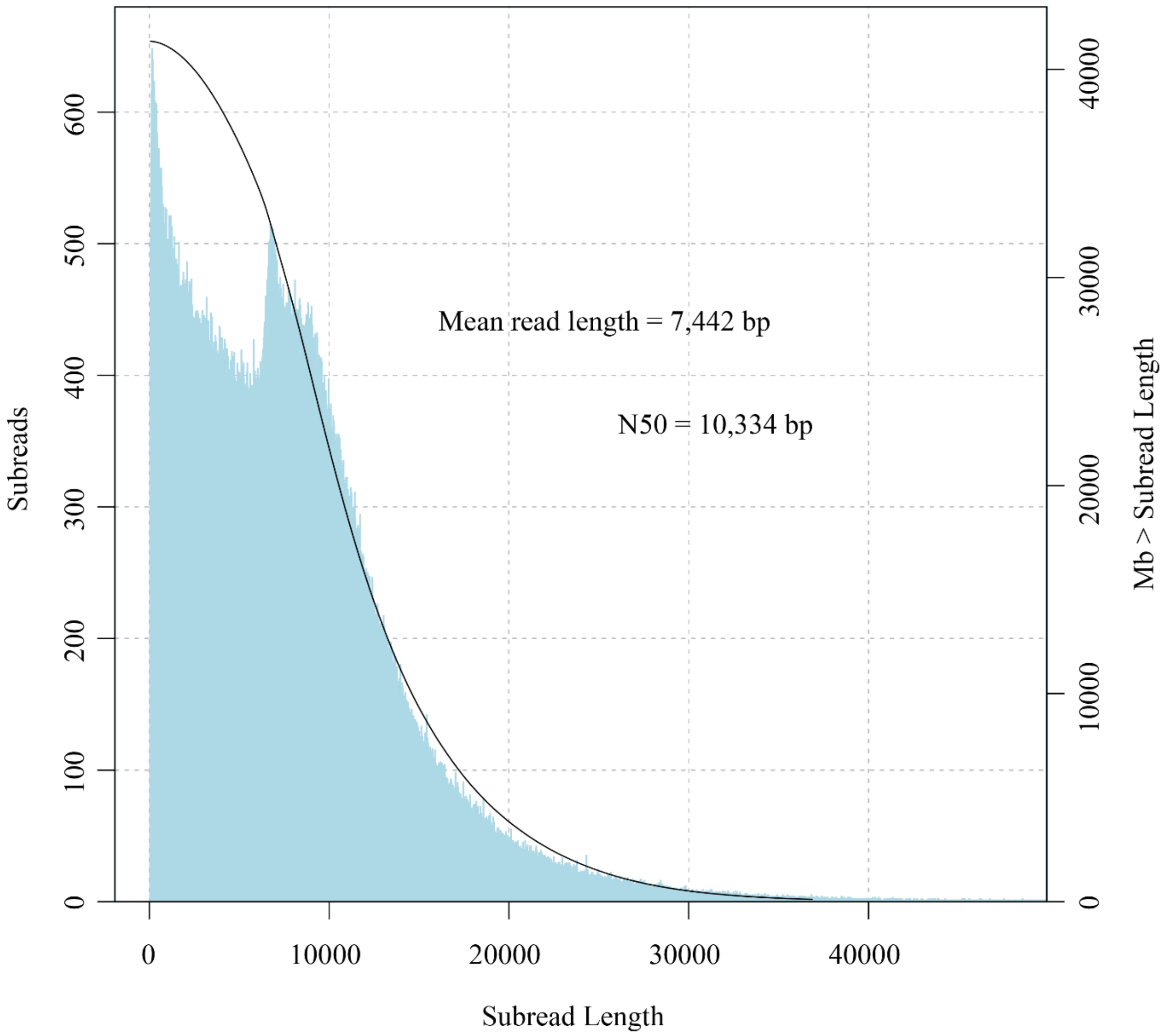

Supplementary Figure 1. Histogram of length distribution of raw *C. acuminata* PacBio subreads. Source data are provided as a Source data file.

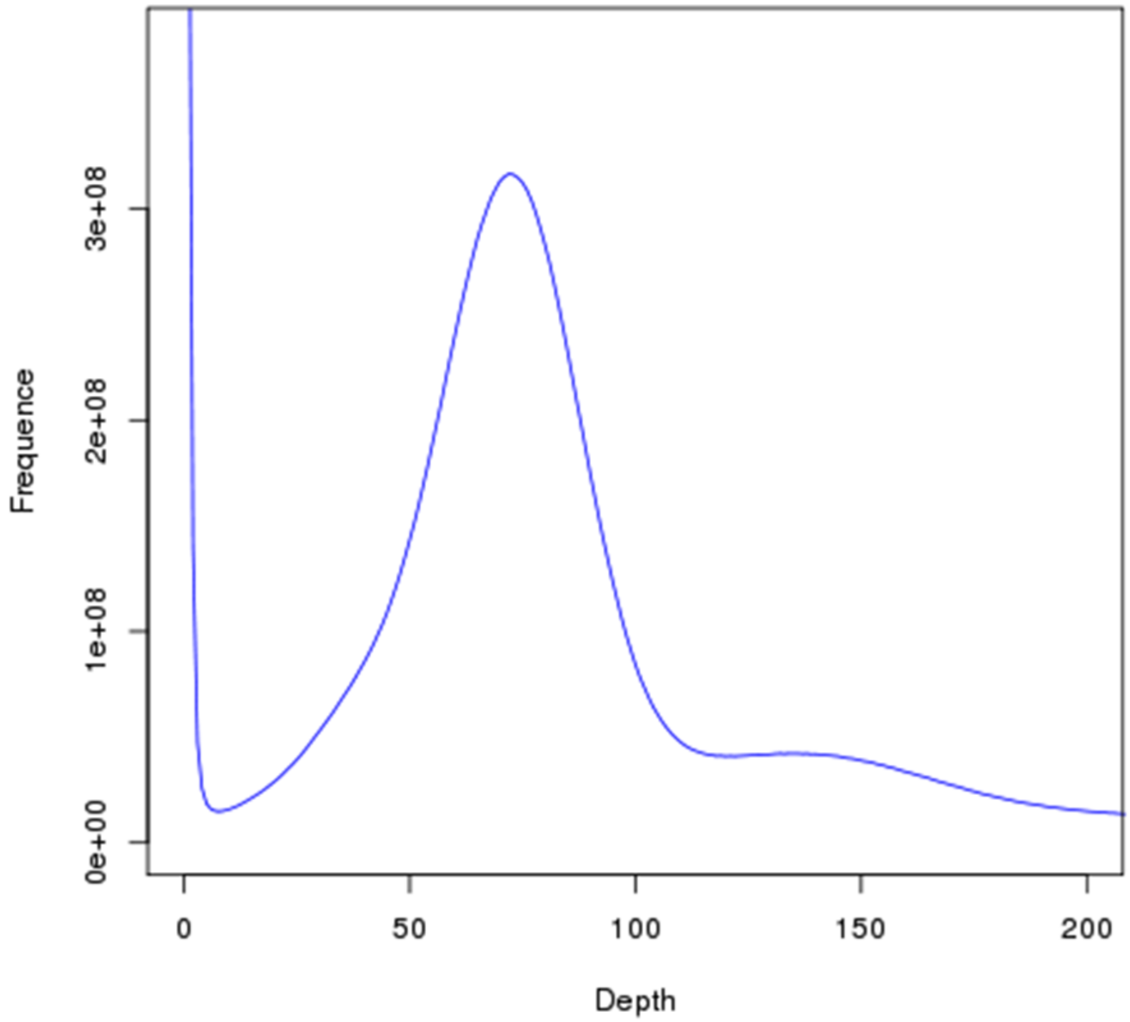

Supplementary Figure 2. Estimation of *C. acuminata* genome size by K-mer analysis. The figure shows the frequency of 17 k-mers, which are 17 bp sequences from clean reads of short-insert-size libraries. We identified 29,283,188,036 K-mers and the peak of K-mer depth is 71. Genome size can be estimated as (total K-mer number) / (the volume peak). The genome size of *C. acuminata* was thus estimated as 412.44 Mb, and the revise genome size is 404.95 Mb.

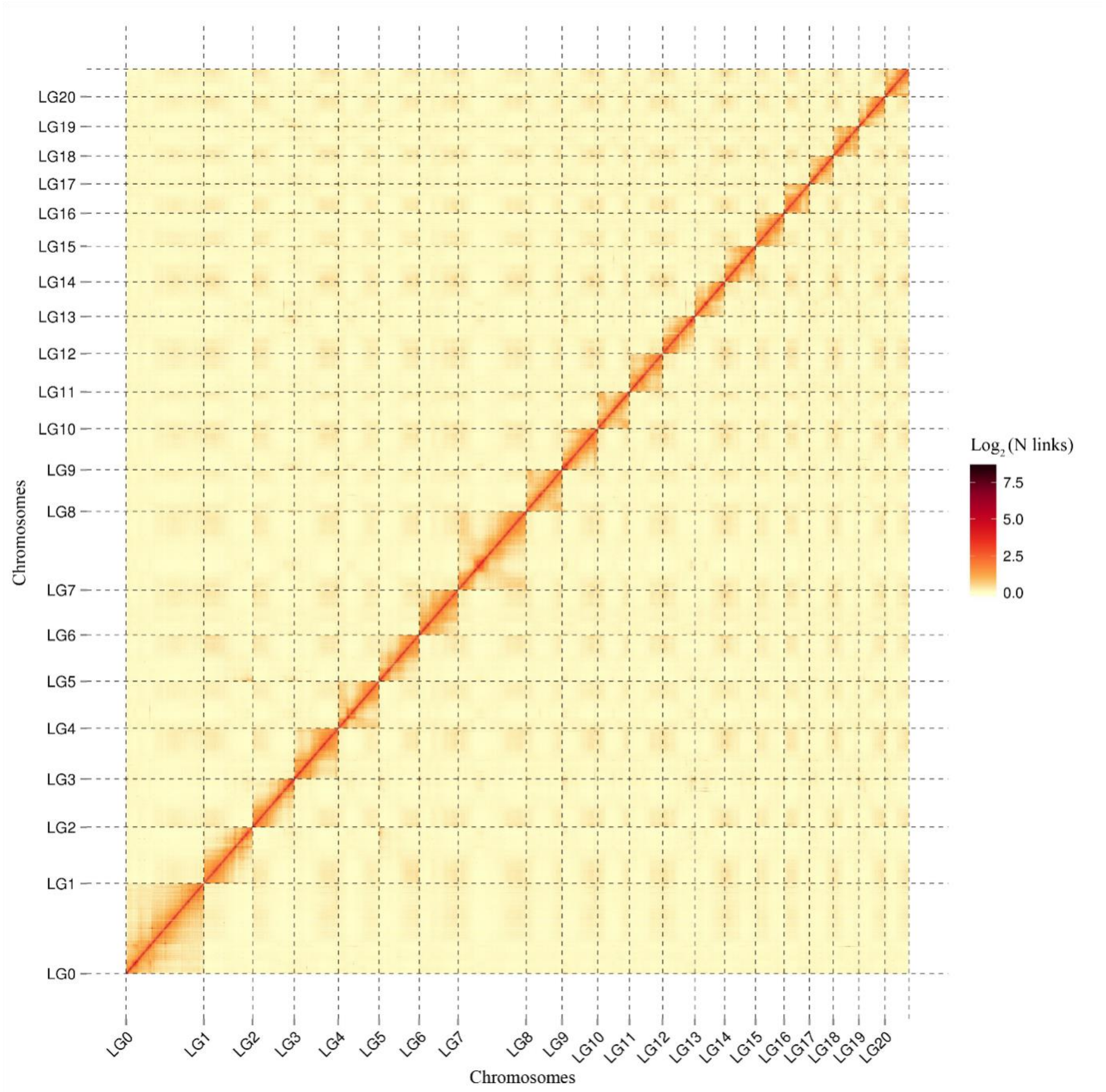

Supplementary Figure 3. Hi-C map of the *C. acuminata* genome showing genome-wide all-by-all interactions. The map shows a high resolution of individual chromosomes that are scaffolded and assembled independently.

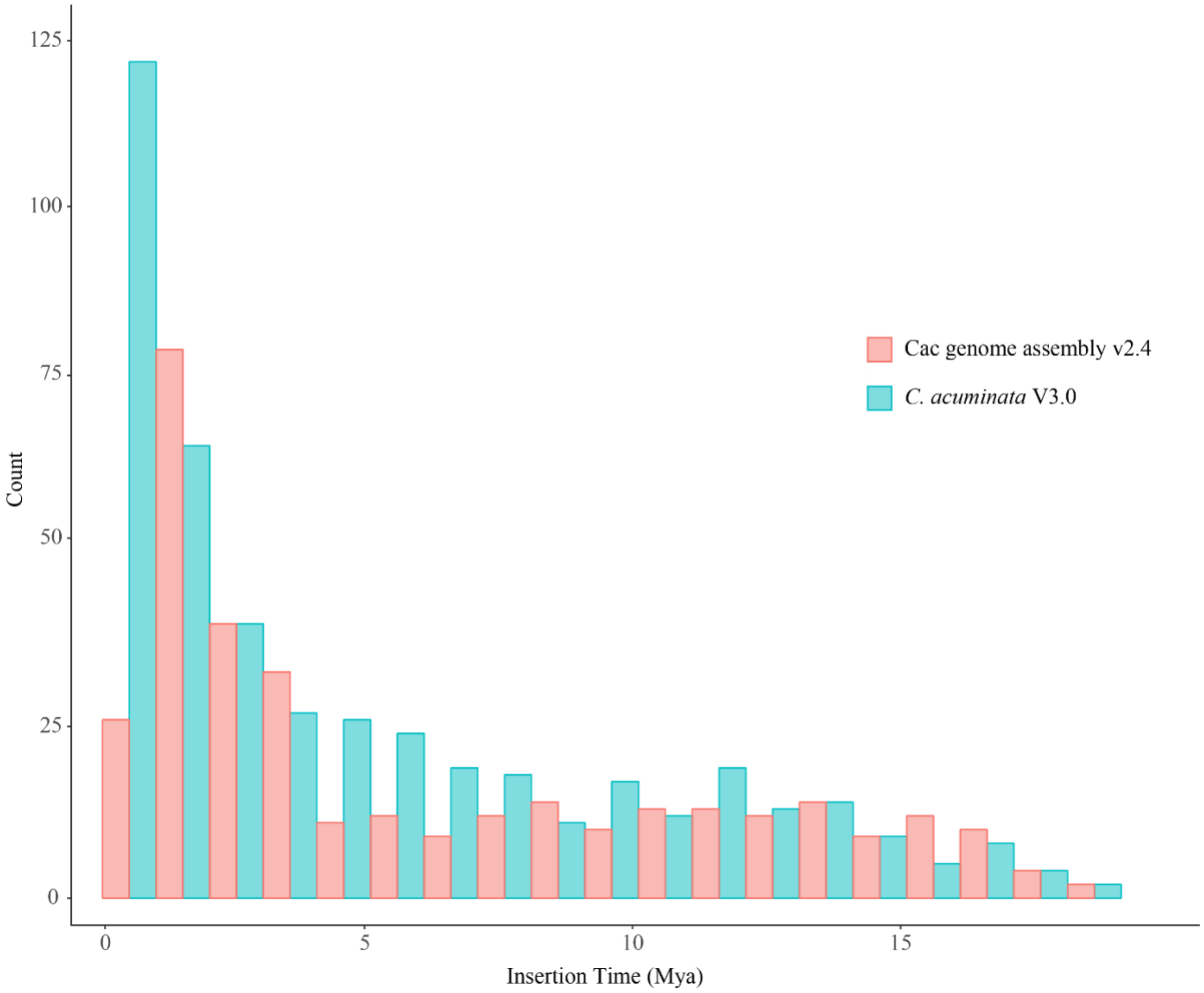

Supplementary Figure 4. The number of intact LTR-RTs birthed at different times (million years ago, Mya) in Cac genome assembly v2.4 and *C. acuminata* V3.0 genome. Source data are provided as a Source data file.

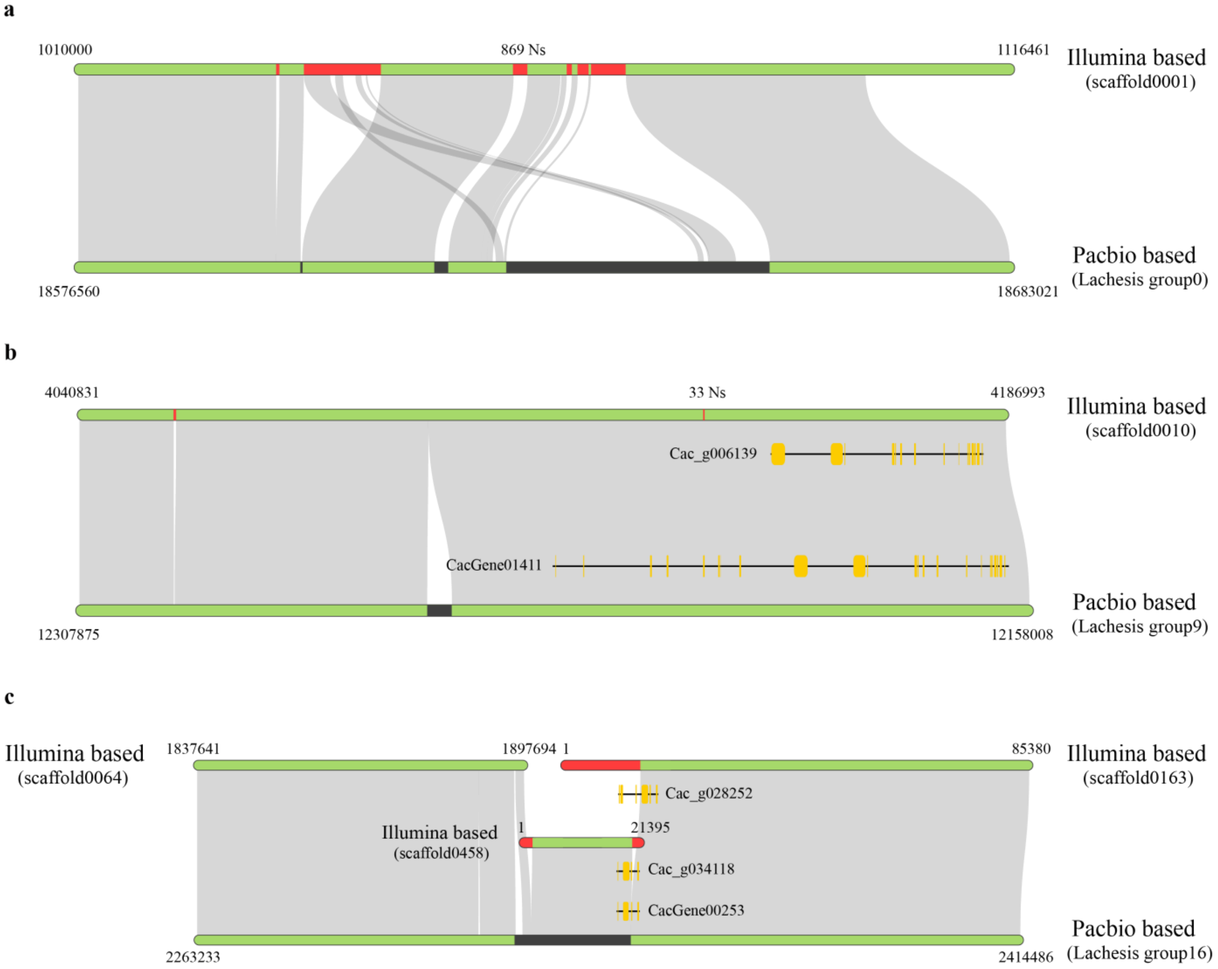

Supplementary Figure 5. Comparison of two *C. acuminata* genome assemblies. **a** Summary comparison of the Illumina-based and PacBio-based *C. acuminata* assemblies, showing the PacBio-based genome filled the gaps and correcting erroneous assembly (black) comparing with the Illumina-based sequences (red). **b-c** Comparison of the Illumina-based and PacBio-based *C. acuminata* assemblies, showing similar corrections that further lead to a correction in the protein-coding sequence prediction (yellow).

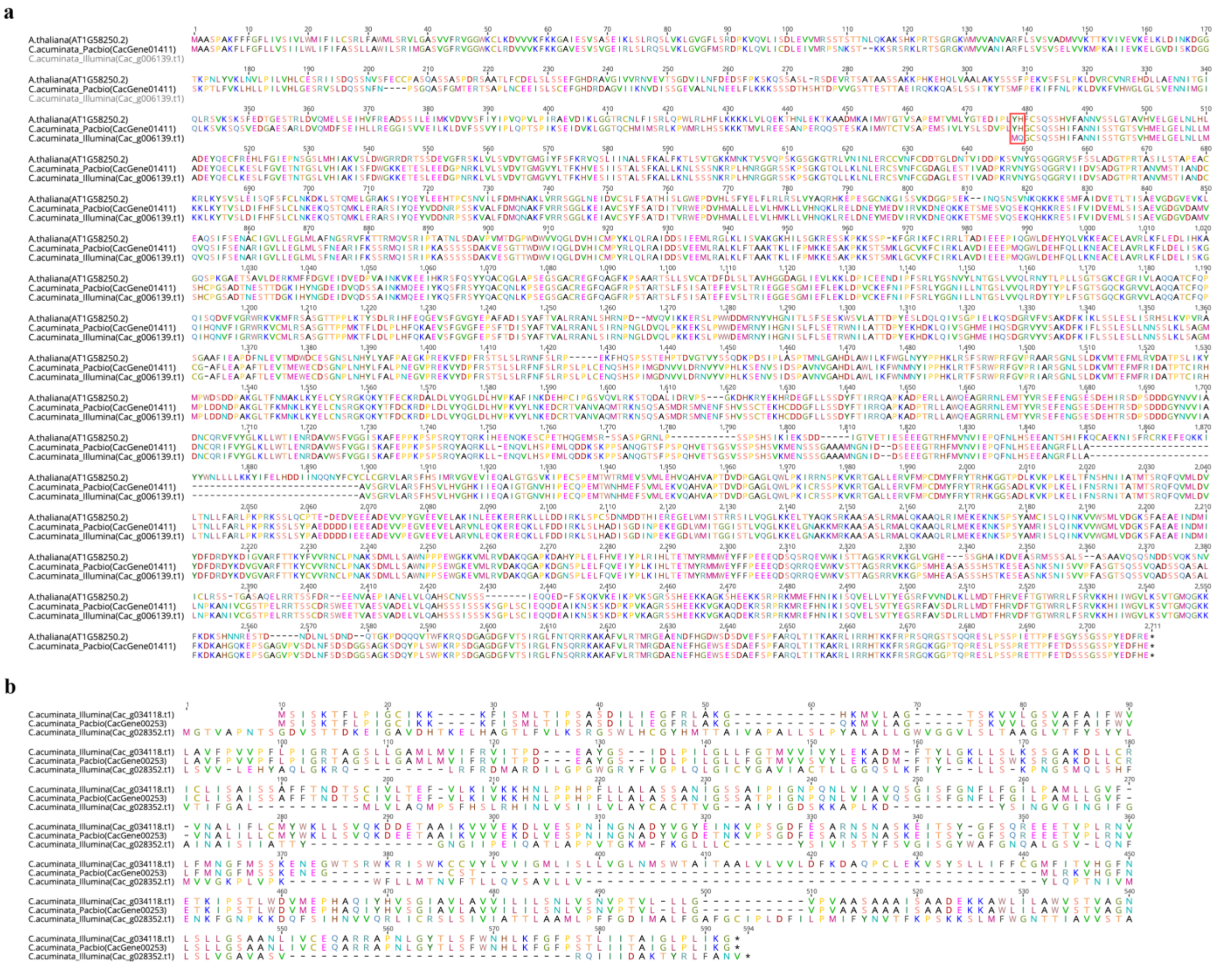

Supplementary Figure 6. Multiple sequence alignment of the proteins for the 2 assemblies in Supplementary Figure 5b and 5c. Showing corrections to the Illumina-based *C. acuminata* protein prediction by the PacBio-based assembly. AT1G58250 in **a** is the homolog gene of CacGene01411 and Cac_g006139.

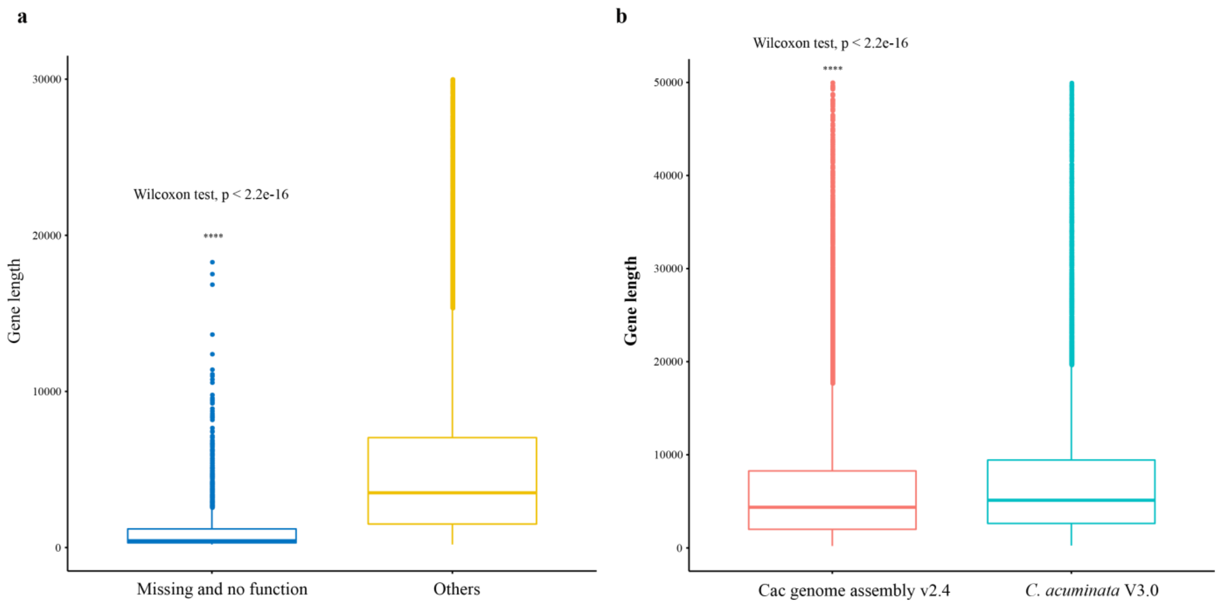

Supplementary Figure 7. Boxplot of protein-coding genes may have wrong structure annotation in Cac genome assembly v2.4 compare with *C. acuminata* V3.0. **a** Possible falsely annotated genes (n = 2,273) compare with other genes (n = 29,588) in Cac genome assembly v2.4 (significance tested by two-tailed Wilcoxon method with W = 9359427, *p*-value = 0 < 2.2e^{-16}); **b** Possible partially annotated genes in Cac genome assembly v2.4 compare with their 1:1 ortholog genes in *C. acuminata* V3.0 (both n = 18,116, significance tested by two-tailed Wilcoxon method with W = 181717587, *p*-value = 3.959343e-70 < 2.2e^{-16}). In **a-b** data are represented as boxplots where the middle line is the median, the lower and upper hinges correspond to the first and third quartiles, the upper whisker extends from the hinge to the largest value no further than 1.5 × IQR from the hinge (where IQR is the inter-quartile range) and the lower whisker extends from the hinge to the smallest value at most 1.5 × IQR of the hinge, while data beyond the end of the whiskers are outlying points that are plotted individually. Source data are provided as a Source data file.

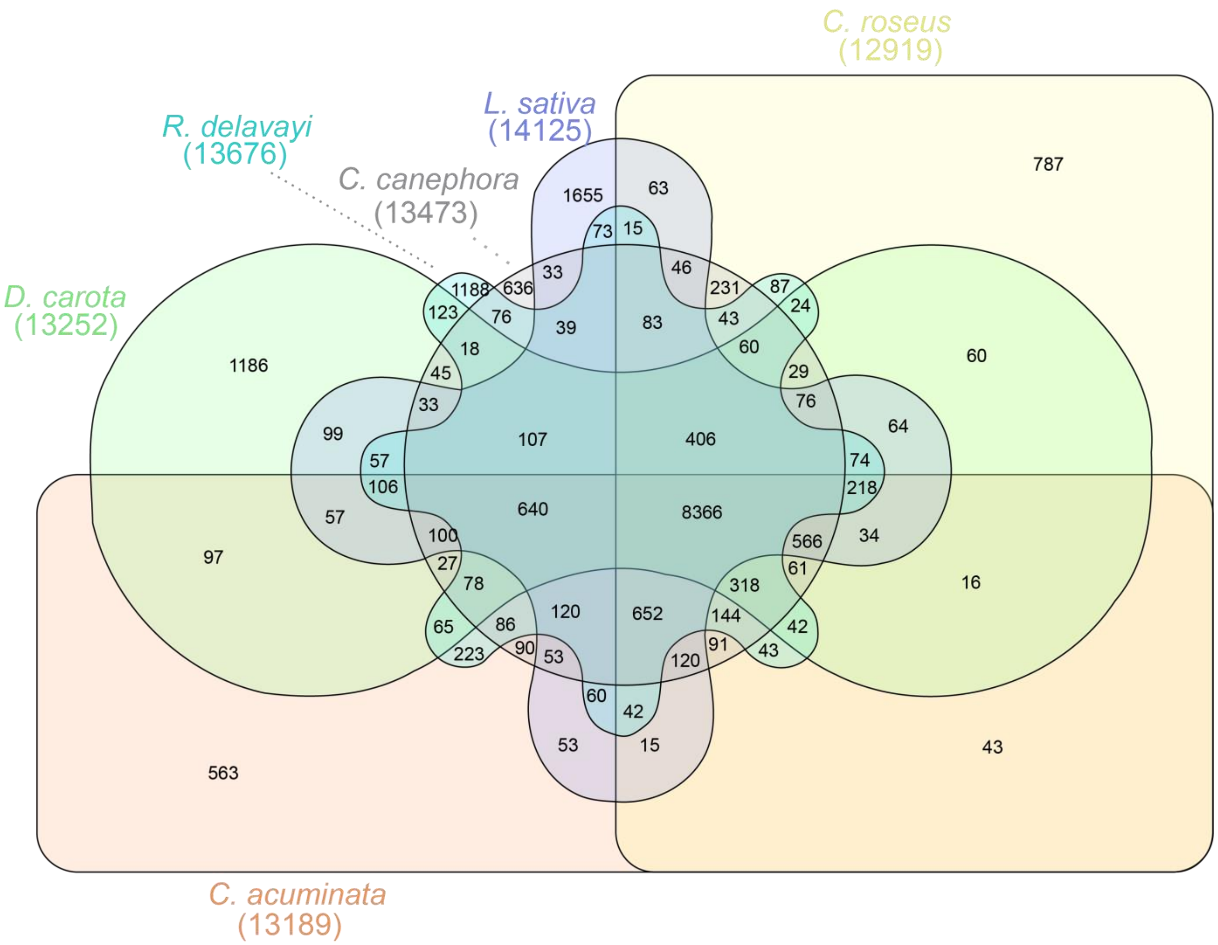

Supplementary Figure 8. Shared and unique gene families. Venn diagram representation of shared/unique gene families among *C. acuminata*, *R. delavayi*, *C. roseus*, *C. canephora*, *D. carota* and *L. sativa*. Only the numbers of gene families that have at least two members from either different species or the same species were shown here. Venn diagram generated by http://www.interactivenn.net/. Source data are provided as a Source data file.

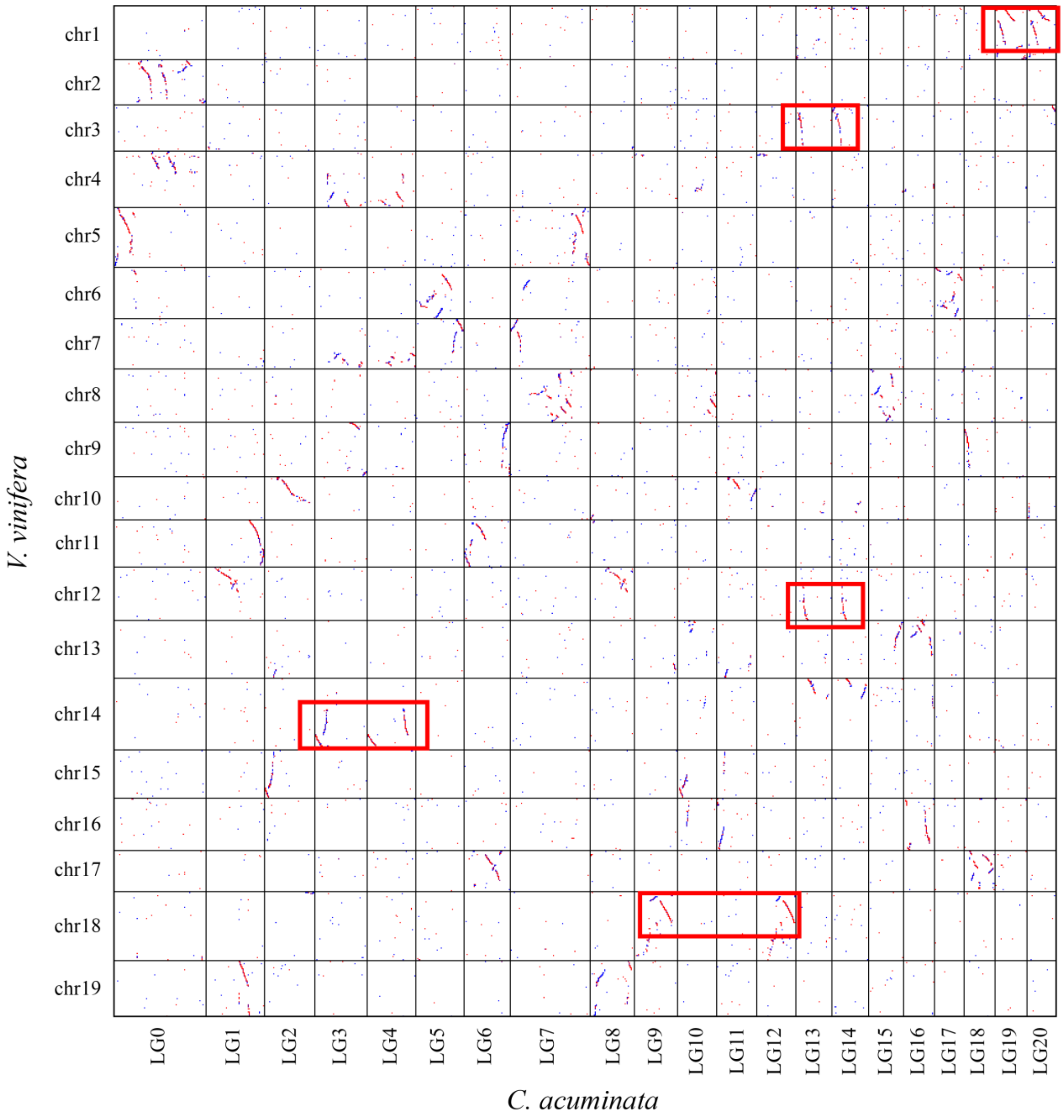

Supplementary Figure 9. Dot plot illustrating the comparative analysis of the *C. acuminata* and *V. vinifera* genomes. The red rectangles highlight several major duplication event, the dots represent the genome sequences' synteny detected by last v963.

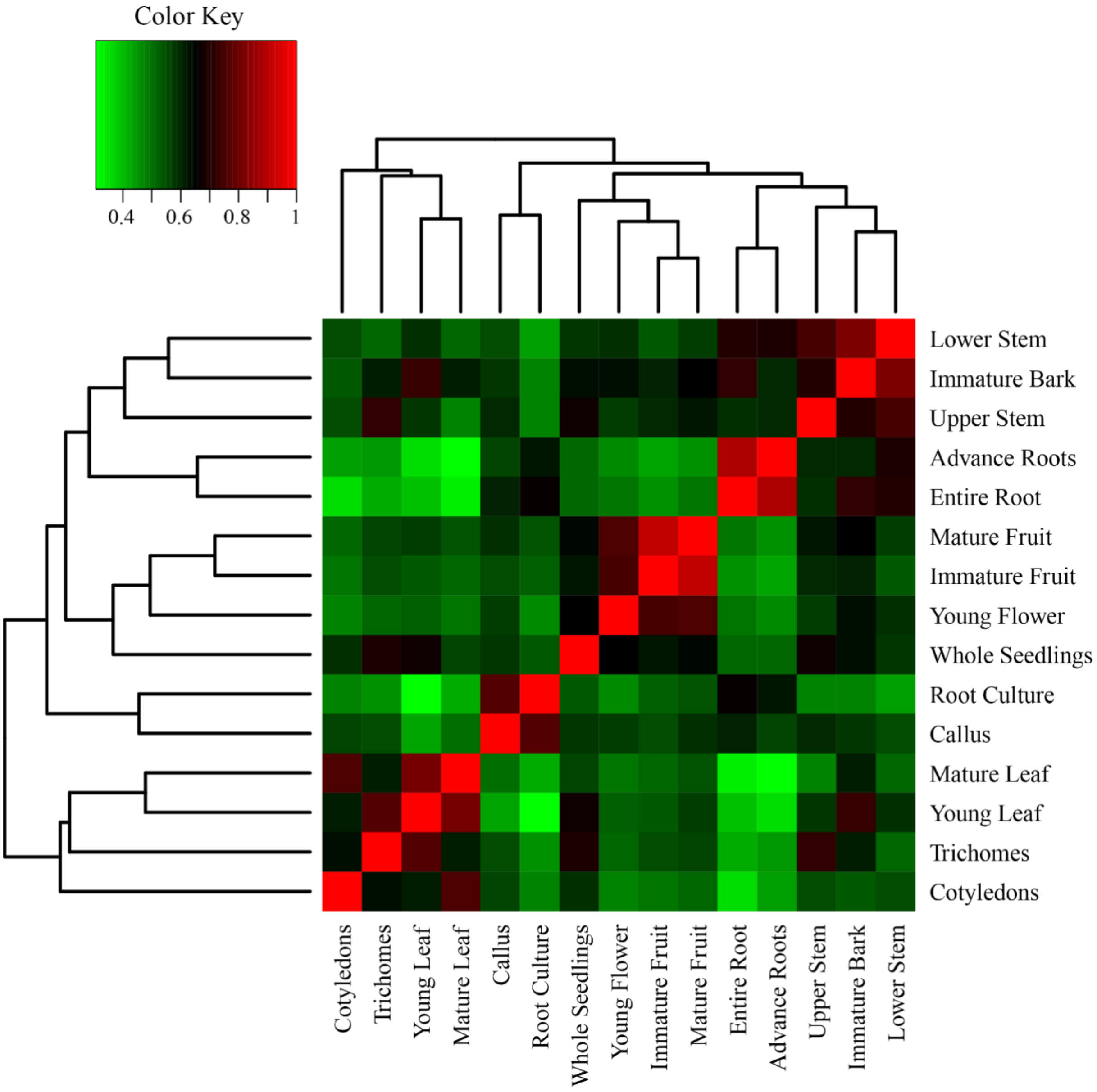

Supplementary Figure 10. Heat-map of differential expression among 15 tissues of *C. acuminata*.

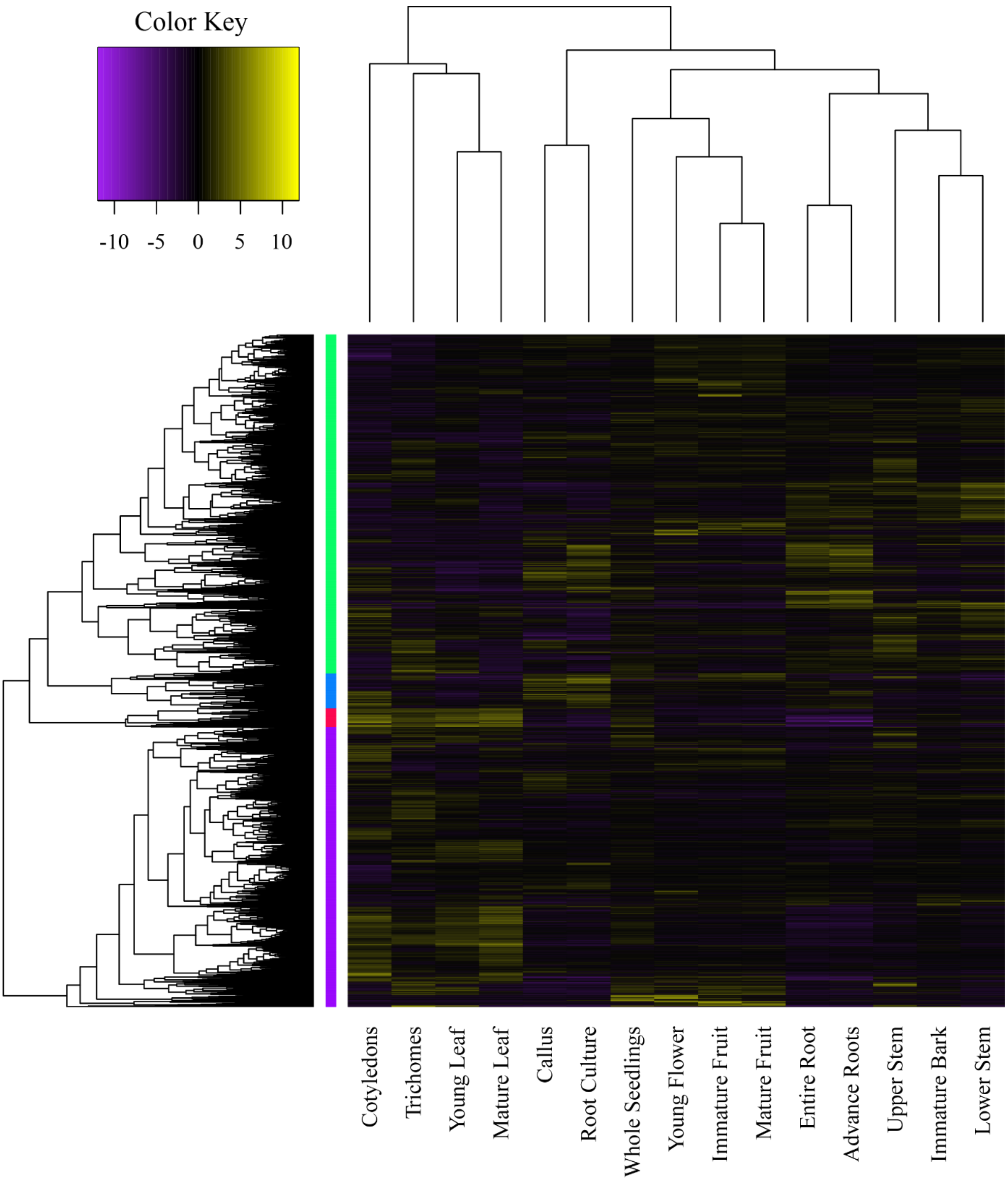

Supplementary Figure 11. Heat-map of differential expressed genes of *C. acuminata*.

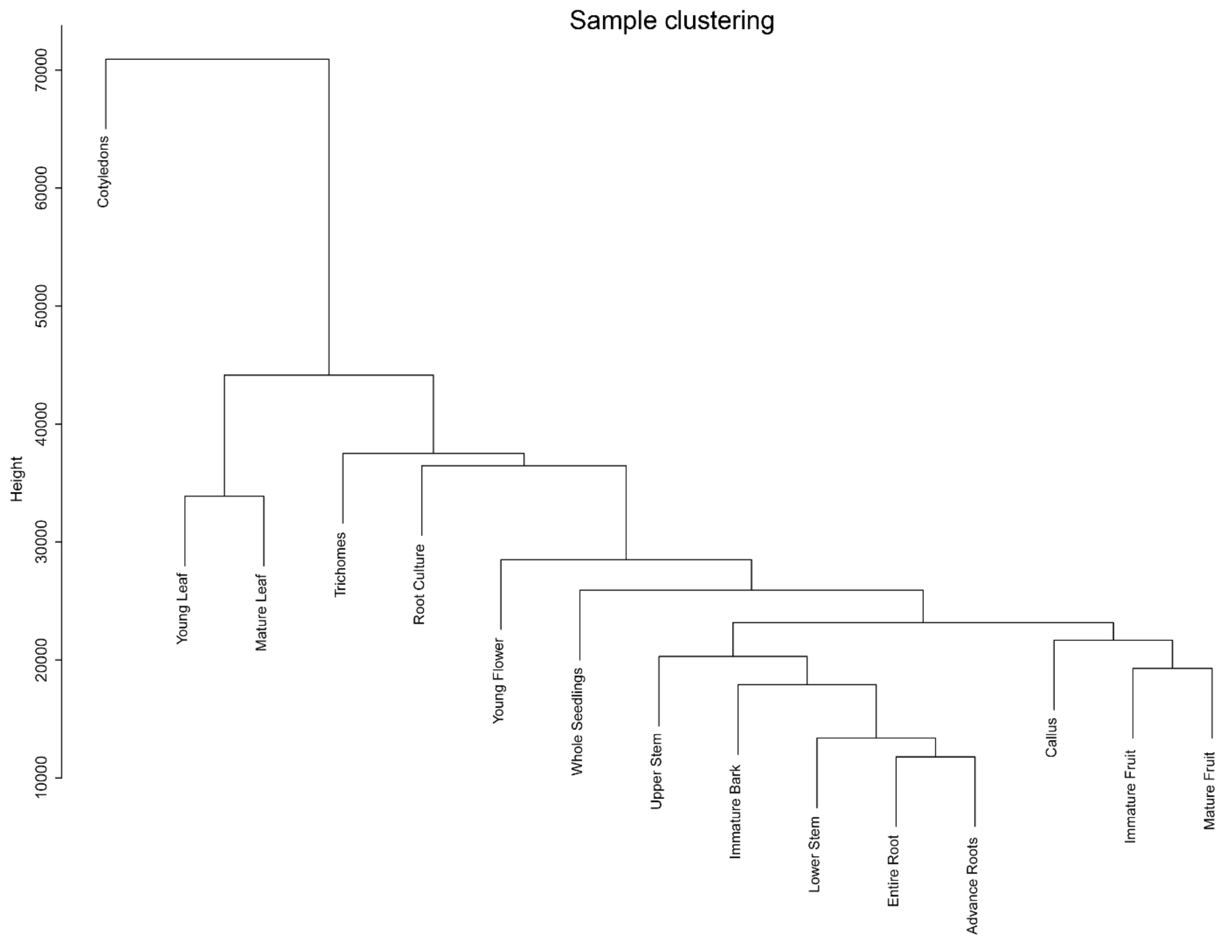

Supplementary Figure 12. Sample clustering for different tissues from WGCNA.

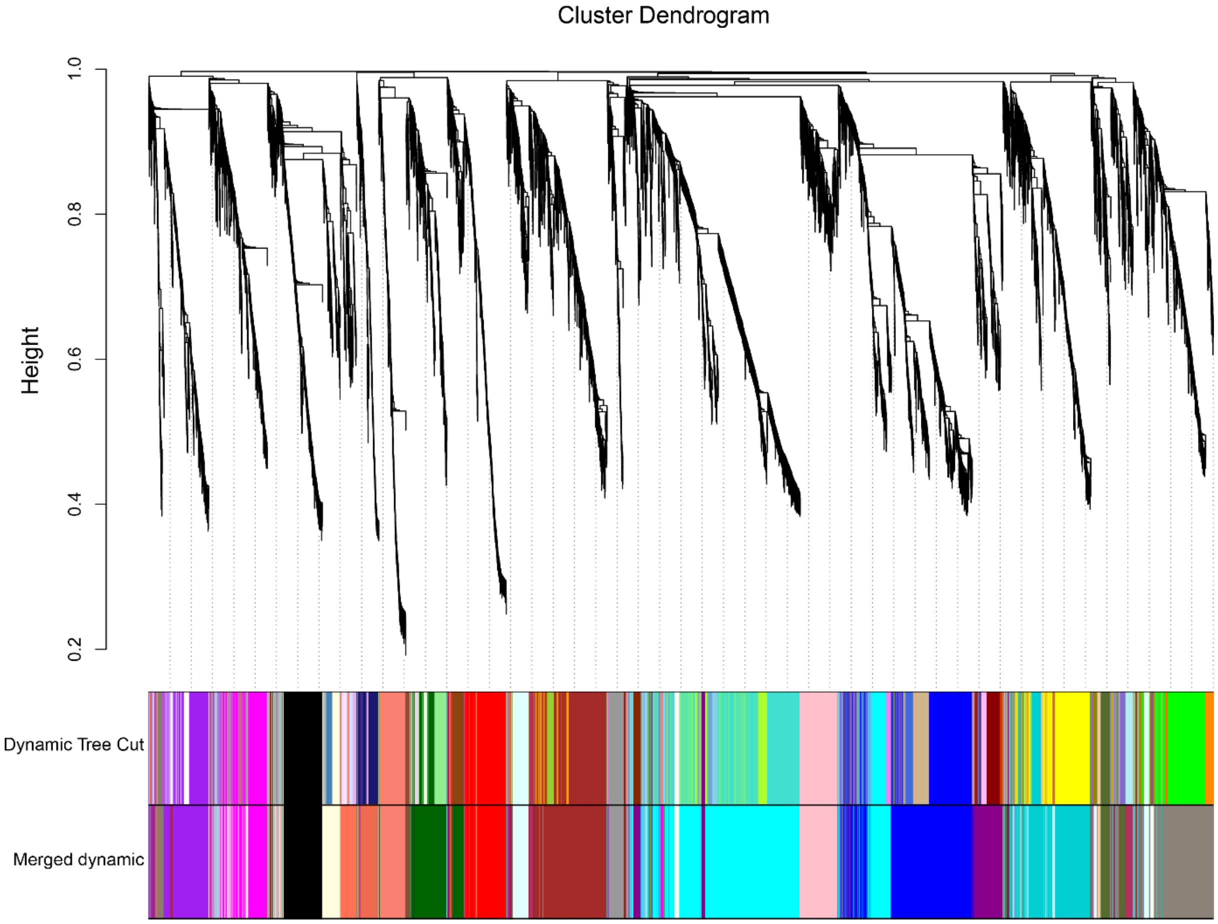

Supplementary Figure 13. Clustering dendrogram of differential expressed genes together with assigned merged module colours and the original module colours. The different colours under the dendrogram show co-expression modules identified using WGCNA. Some key genes in indole alkaloid biosynthetic pathway were assigned to the module with grey60 color.

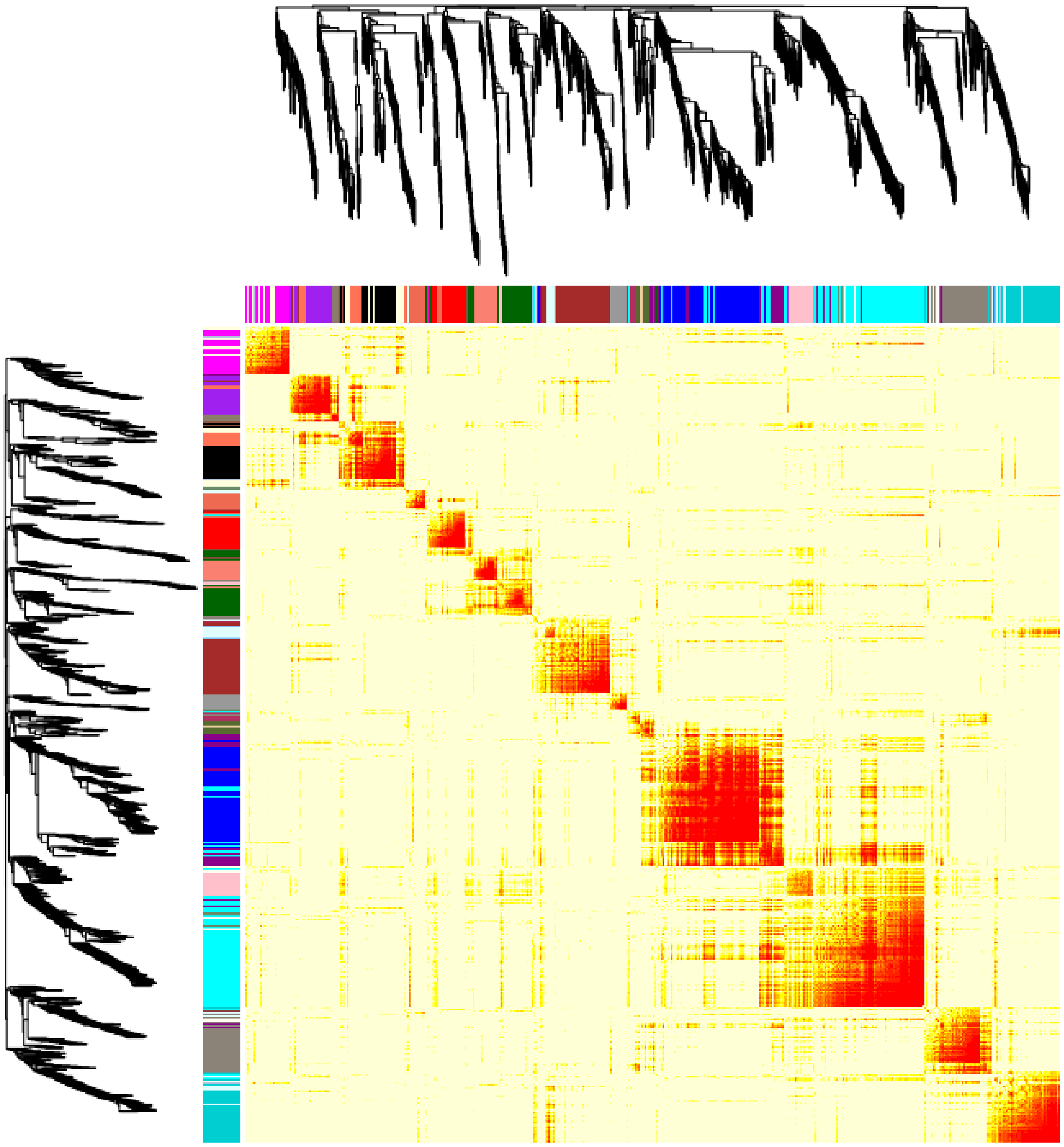

Supplementary Figure 14. Heatmap of the gene network using Topological Overlap Matrix among all differential expressed genes identified from different tissues. The left side and the top represent the gene dendrogram and module. The colour bar that next to the dendrogram shows the co-expressed modules.

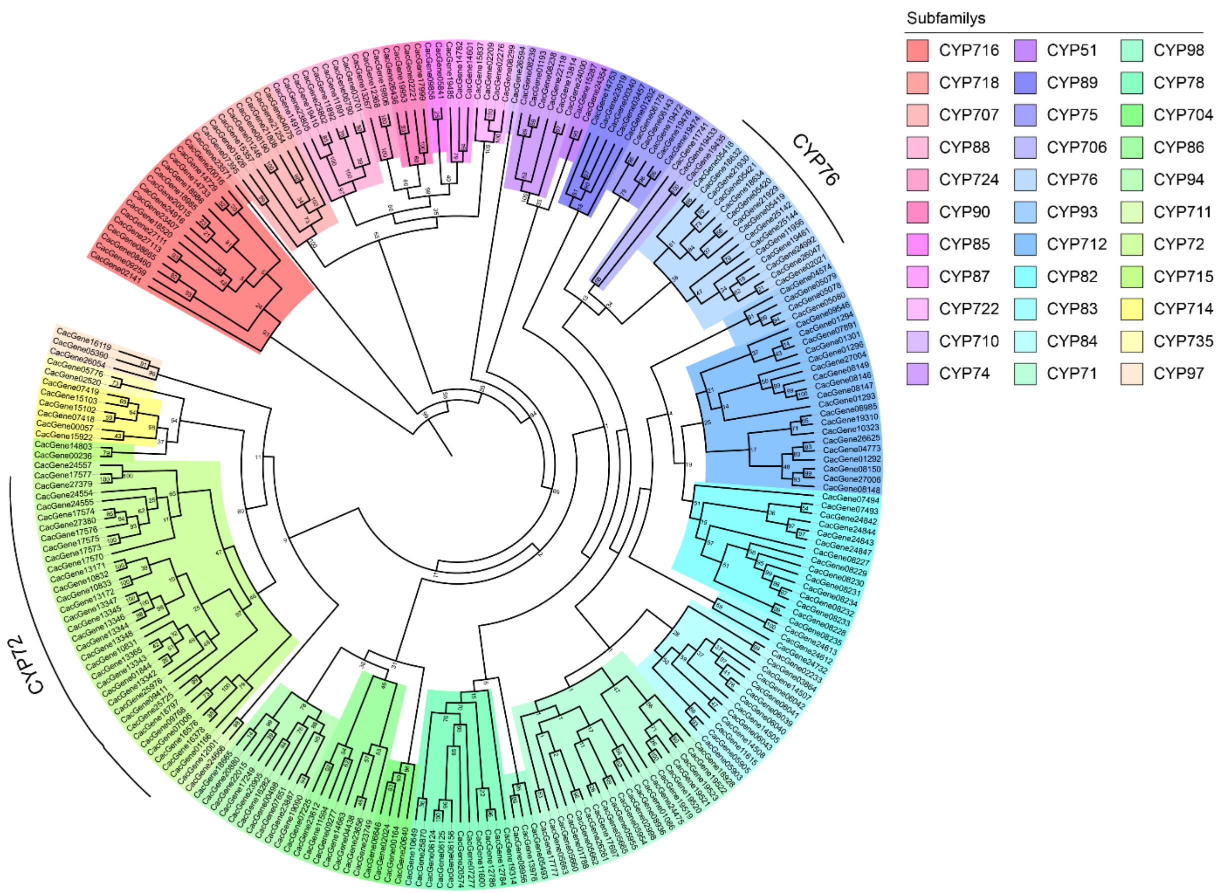

Supplementary Figure 15. Phylogenetic tree of the candidate CYP450 enzyeme encoding genes identified in *C. acuminata* genome. Different colors indicate different CYP450 family members classified based on the protein domain annotation, homolog searching by *A. thaliana* and SwissProt protein database. Families which confirming involved in camptothecin biosynthesis previously were marked by arcs.

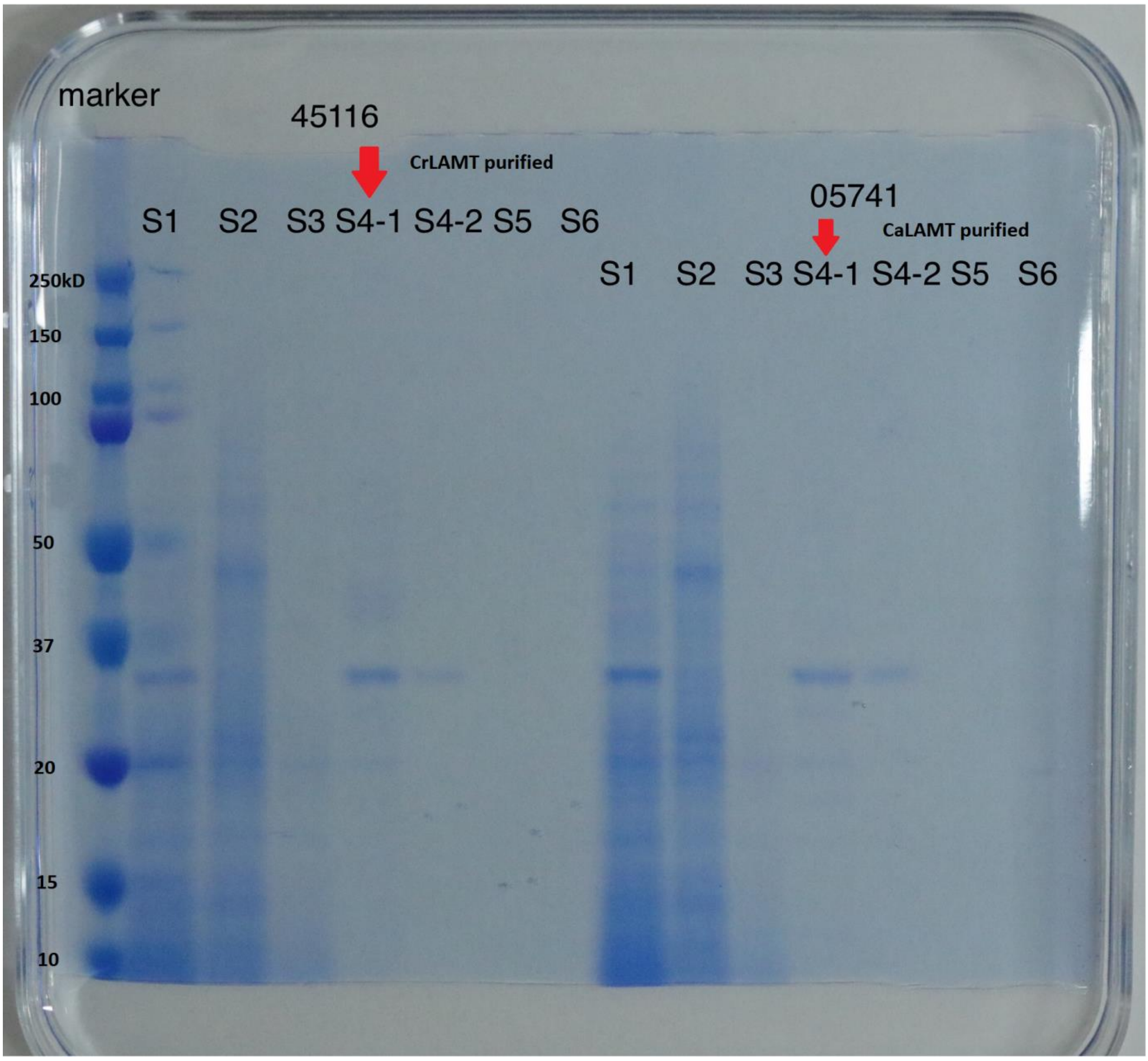

Supplementary Figure 16. SDS-PAGE of purified CrLAMT and CaLAMT. Line of the two purified proteins were highlighted by the red arrows. The experiment was repeated with similar results in line S4-1 and S4-2 respectively.

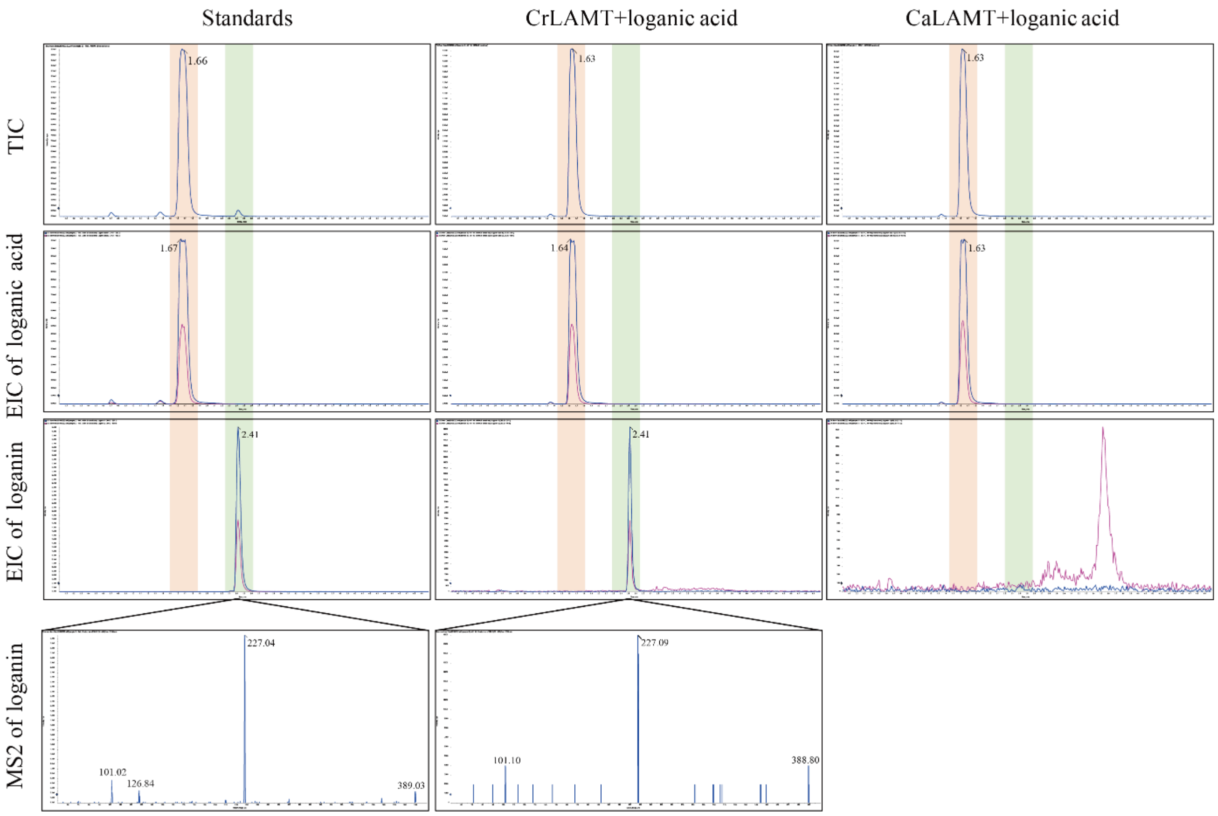

Supplementary Figure 17. Conversion reactions from loganic acid to loganin by CrLAMT and CaLAMT. TIC: Total Ion Chromatography, EIC: extracted ion chromatography. For MS2 model, the daughter ions of loganin (m/z, 389.2) was scanned between 50-400.

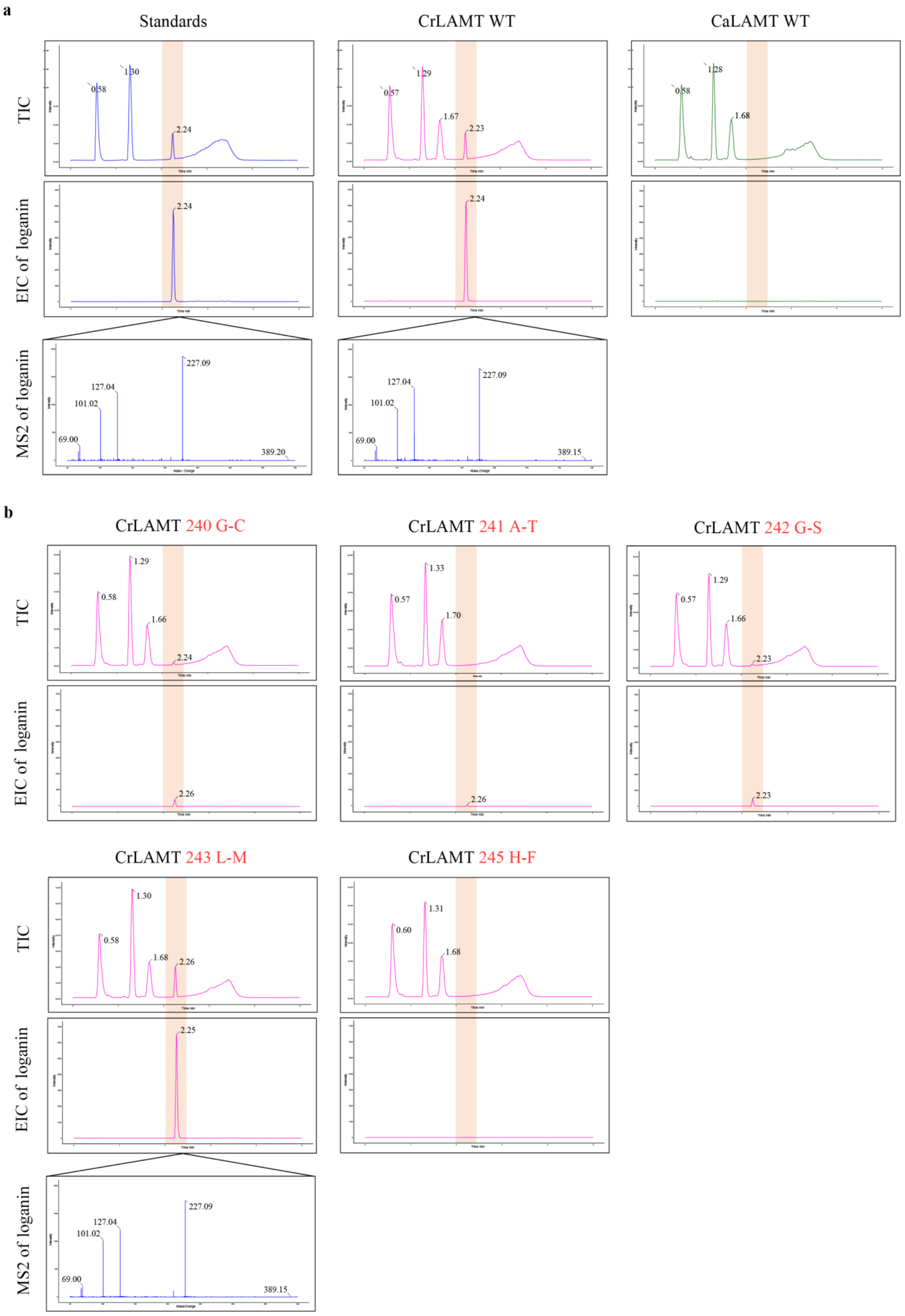

Supplementary Figure 18. Conversion reactions from loganic acid to loganin by CrLAMT WT, CaLAMT WT (**a**) and the CrLAMT mutates (**b**). TIC: Total Ion Chromatography, EIC: extracted ion chromatography. For MS2 model, the daughter ions of loganin (m/z, 389.2) was scanned between 50-400.

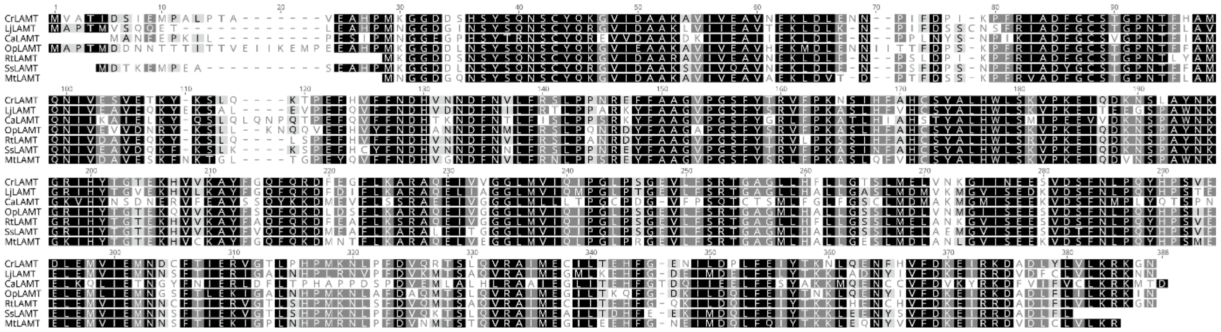

Supplementary Figure 19. Sequence alignment of LAMT encoding gene copies in *C. acuminata* and other species.

a

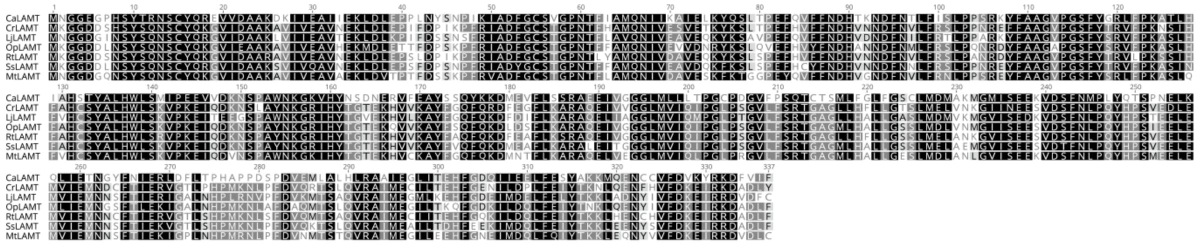

b

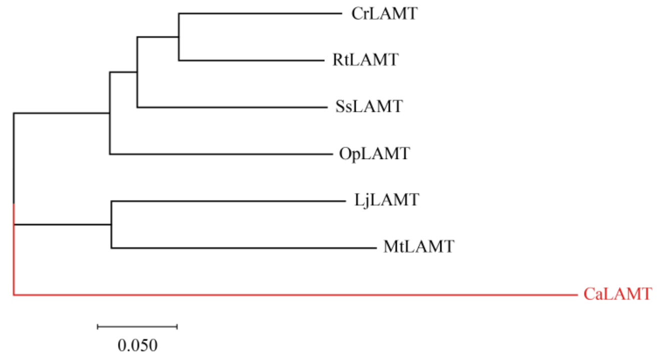

Supplementary Figure 20. Trimmed sequence alignment (**a**) and unrooted tree (**b**) used for selection analyses with LAMT encoding gene copies in *C. acuminata* and other species. Red indicate the foreground branches.

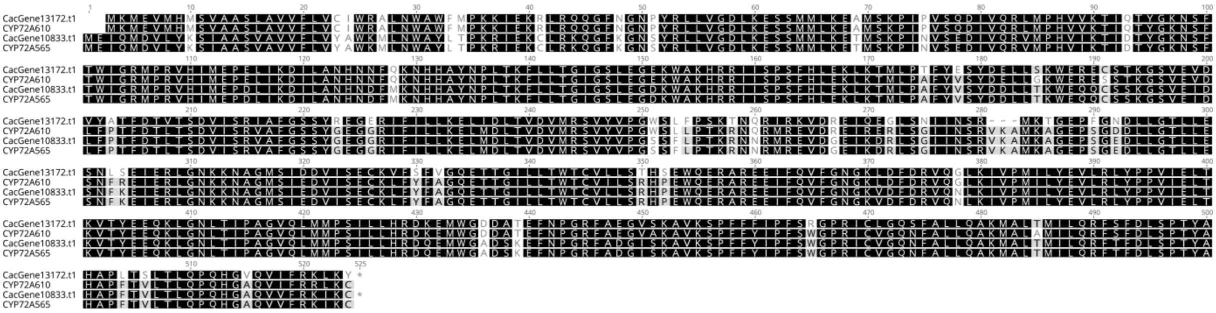

Supplementary Figure 21. Sequence alignment of SLAS CYP72A565 and CYP72A610 with their corresponding protein in *C. acuminata* genome assembly V3.0.

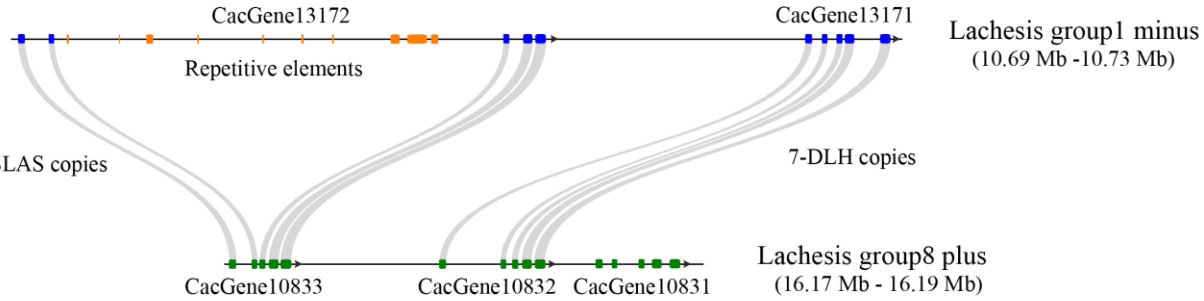

Supplementary Figure 22. Location and collinear relationship of 7-DLH and SLAS encoding gene copies in *C. acuminata* genome assembly V3.0.

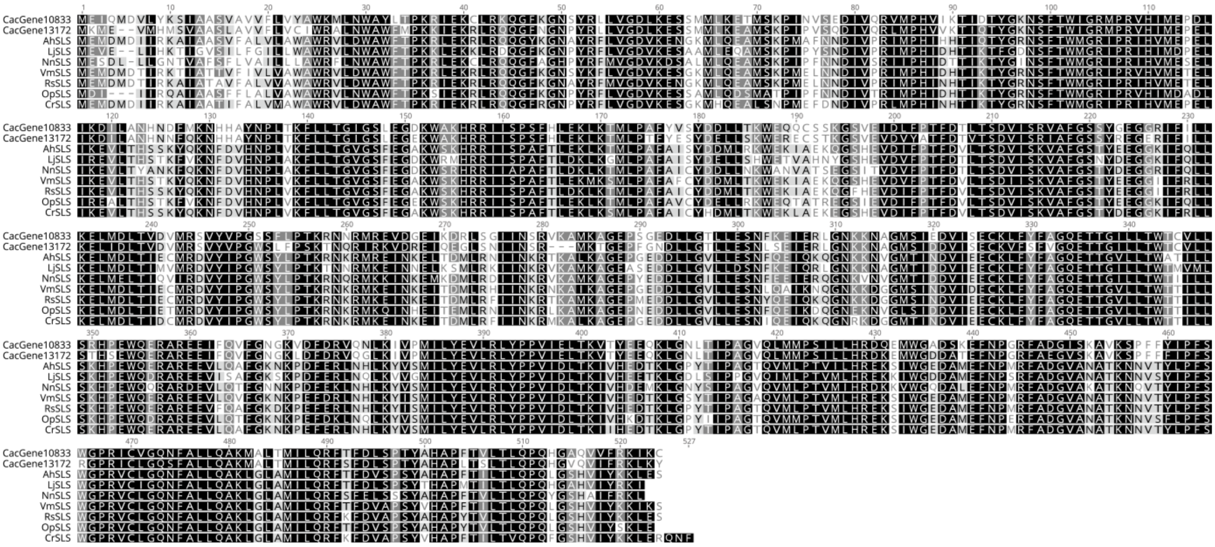

Supplementary Figure 23. Sequence alignment of SLAS encoding gene copies in *C. acuminata* with SLS-like encoding genes in other species.

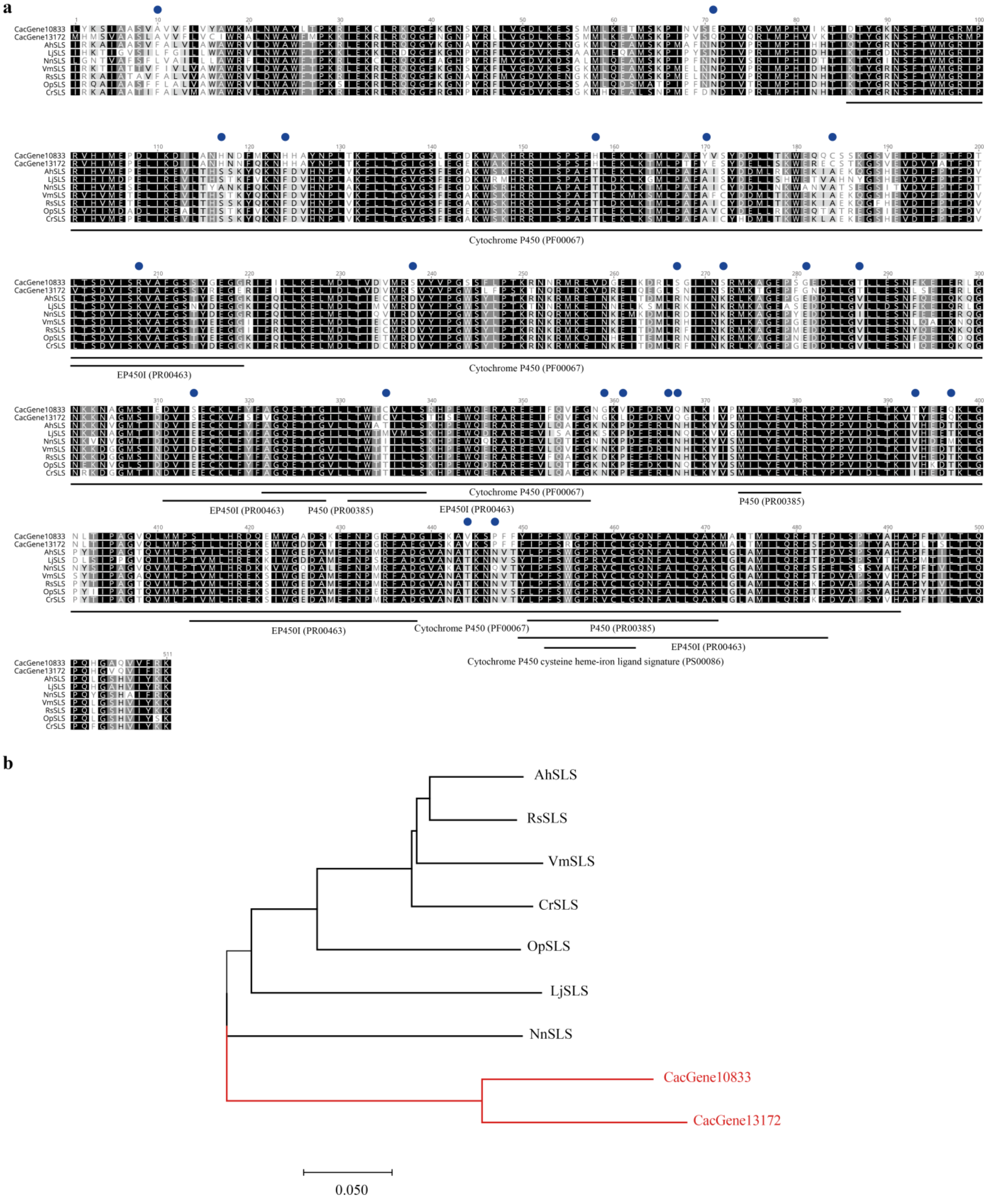

Supplementary Figure 24. Trimmed sequence alignment (**a**) and unrooted tree (**b**) used for selection analyses in SLAS encoding gene copies in *C. acuminata* and SLS-like encoding genes in other species. Red indicate the foreground branches. Positive sites were shown in blue.

a

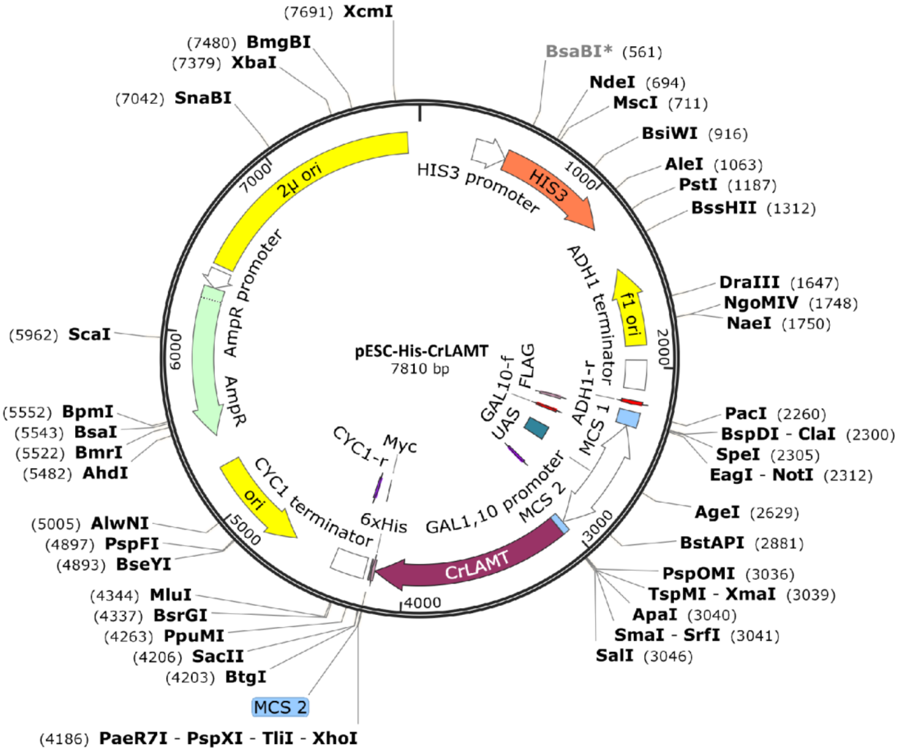

b

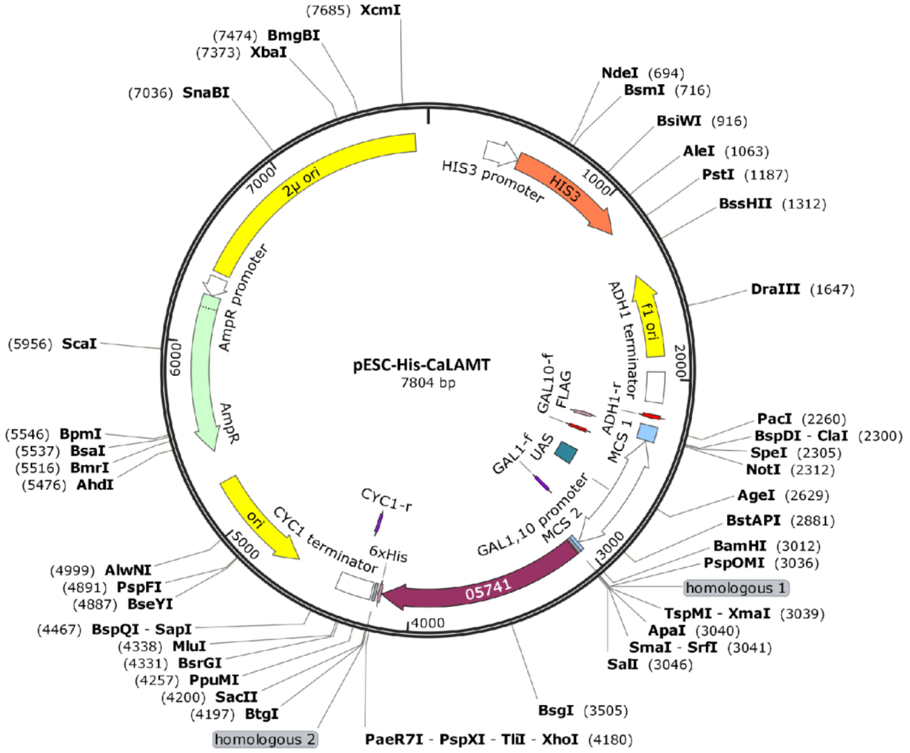

Supplementary Figure 25. pESC-His expression vector with His tag of (**a**) CrLAMT and (**b**) CaLAMT.

Supplementary Table 1. Summary of DNA sequencing data.

Read type	Number of clean reads / subreads	Insert size (bp)	Total data (Gb)	Read length / Mean subread length (N50) (bp)	Sequence coverage (X) [1]
Illumina pair-end reads	158,859,043	350	47.66	2 x 150	118.18
Hi-C pair-end reads	197,679,512	300	59.30	2 x 150	146.45
Pacbio subreads	5,556,300	-	41.35	7,442 (10,334)	102.11

[1]Depth was calculated under the estimate of a genome size of 404.95Mb.

Supplementary Table 2. Statistics of reads mapping rate and coverage of *C. acuminata* V3.0 genome assembly.

		Percentage (%)
Reads	Mapping rate	94.84
Genome	Coverage	95.71
	Coverage at least 4X	91.31
	Coverage at least 10X	86.62
	Coverage at least 20X	80.85

Supplementary Table 3. K-mer analysis of the *C. acuminata* V3.0 genome by using K-mer=17.

K-mer	K-mer number	K-mer depth (X)	Genome size（Mb）	Revised genome size (Mb)	Heterozygous ratio(%)	Repeat(%)
17	29,283,188,036	71	412.44	404.95	0.89	45.20

Supplementary Table 4. Statistics of *C. acuminata* V3.0 genome after assembly by Hi-C data.

Group	Sequence number[1]	Sequence length (bp)[1]
Lachesis Group 0	63	40,362,105
Lachesis Group 1	35	24,922,017
Lachesis Group 2	43	22,233,934
Lachesis Group 3	16	22,176,898
Lachesis Group 4	42	21,584,715
Lachesis Group 5	42	21,155,450
Lachesis Group 6	24	19,901,506
Lachesis Group 7	38	34,781,413
Lachesis Group 8	33	18,845,215
Lachesis Group 9	21	18,359,844
Lachesis Group 10	50	17,246,343
Lachesis Group 11	25	17,184,261
Lachesis Group 12	26	16,630,491
Lachesis Group 13	28	15,902,045
Lachesis Group 14	12	15,714,743
Lachesis Group 15	41	15,587,416
Lachesis Group 16	45	14,216,941
Lachesis Group 17	52	14,042,992
Lachesis Group 18	36	13,553,910
Lachesis Group 19	14	13,406,917
Lachesis Group 20	20	12,444,790
Total Sequences Clustered (%)	706 (62.48)	410,253,946 (98.88)
Total Sequences Ordered and Oriented (%)	376 (53.26)	393,985,980 (96.03)

[1]Sequence number and length was calculated with all clustered sequences > 1 Kb.

Supplementary Table 5. Benchmarking Universal Single-Copy Orthologs (BUSCO) analysis about Cac genome assembly v2.4 and *C. acuminata* V3.0.

	Total BUSCO groups searched	Complete single-copy BUSCOs	Complete duplicated BUSCOs	Fragmented BUSCOs	Missing BUSCOs	Complete BUSCOs percentage (%)
Cac genome assembly v2.4	1440	1244	104	36	56	93.6
C. acuminata V3.0	1440	1270	97	21	52	94.9

Supplementary Table 6. Intact LTR-RTs identified in Cac genome assembly v2.4 and *C. acuminata* V3.0.

	Total intact LTR-RTs searched	Copia	Gypsy	Unknown
Cac genome assembly v2.4	340	180	65	95
C. acuminata V3.0	465	208	63	194

Source data are provided as a Source data file.

Supplementary Table 7. Classification of repetitive elements in the *C. acuminata* V3.0.

Type	Repeat size (bp)	number of elements	% of genome
DNA	28,477,637	114,478	6.86
LINE	19,209,794	41,079	4.63
SINE	1,114,895	7,584	0.27
LTR	56,029,493	74,750	13.50
Unclassified	50,997,452	214,155	12.29
Satellite	47,670	321	0.01
Simple repeats	494,387	2,623	0.12
Total interspersed repeats[1]	155,829,271	452,046	37.55
Total	156,371,328	454,990	37.68

[1]Repetitive elements for masking genome for gene prediction, expect Satellite and Simple repeats. Because these type of repetitive elements are of short length, the probability of appearing in the coding region is high.

Supplementary Table 8. Comparison of predicted protein-coding genes between Cac genome assembly v2.4 and *C. acuminata* V3.0.

	**Cac genome assembly v2.4**	***C. acuminata* V3.0**
Number of protein-coding gene	31,825	27,940
Number of transcripts	40,332	51,735
Average transcript length (bp)	6026.33	8394.99
Average exon length (bp)	286.32	269.81
Average intron length (bp)	1008.03	1052.31
Average number of exons per gene	5.07	6.14
Average exon length per gene (bp)	1450.68	1656.63

Supplementary Table 9. Summary of functional annotation of predicted genes for *C. acuminata* V3.0.

	**Numbers of matching genes**	**Percent of annotated genes (%)**
Total	27,940	100
SwissProt	22,121	79.17
InterPro	26,804	95.93
GO	24,691	88.38
TrEMBL	26,036	93.19
KEGG Pathway	9,320	33.36
Annotated[1]	27,122	97.07
Unannotated	818	2.93

[1]At least one match in either of database above.

Supplementary Table 10. Summary of gene family clustering.

Species	Total genes	Genes in families	Family	Unclustered genes	Unique families	Genes per family
A. thaliana	27,416	23,178	12,561	4,238	825	1.85
C. acuminata	27,940	23,047	13,188	4,893	413	1.75
C.roseus	33,829	27,640	12,919	6,189	728	2.14
C.canephora	25,574	20,890	13,473	4,684	537	1.55
D.carota	32,113	26,088	13,252	6,025	1105	1.97
L.sativa	38,910	30,339	14,125	8,571	1593	2.15
R.delavayi	32,938	25,391	13,676	7,547	1104	1.86
V.vinifera	26,346	19,275	12,636	7,071	627	1.53

Source data are provided as a Source data file.

Supplementary Table 11. GO enrichment analysis of species-specific genes in the *C. acuminata* genome. Significance was tested by two tailed Fisher's exact test method.

**GO ID**	**GO name**	**GO category**	**FDR**	***p*-value**	**Number of genes**
GO:0015462	ATPase-coupled protein transmembrane transporter activity	MF	4.44E-32	4.43E-36	29
GO:1902039	negative regulation of seed dormancy process	BP	2.03E-21	4.06E-25	26
GO:0004252	serine-type endopeptidase activity	MF	5.03E-21	1.51E-24	48
GO:0010205	photoinhibition	BP	3.40E-17	1.70E-20	26
GO:0010360	negative regulation of anion channel activity	BP	6.57E-17	4.26E-20	26
GO:0048838	release of seed from dormancy	BP	3.21E-16	2.40E-19	26
GO:0015074	DNA integration	BP	9.35E-16	7.46E-19	35
GO:0009939	positive regulation of gibberellic acid mediated signaling pathway	BP	1.20E-14	1.08E-17	26
GO:0043531	ADP binding	MF	2.02E-14	2.01E-17	66
GO:0009658	chloroplast organization	BP	2.74E-11	4.10E-14	68
GO:0002238	response to molecule of fungal origin	BP	3.26E-11	5.05E-14	42
GO:0043952	protein transport by the Sec complex	BP	3.68E-11	5.88E-14	10
GO:0002230	positive regulation of defense response to virus by host	BP	7.09E-10	1.17E-12	27
GO:0015066	alpha-amylase inhibitor activity	MF	5.95E-07	1.33E-09	9
GO:0009626	plant-type hypersensitive response	BP	9.81E-07	2.25E-09	63
GO:1902456	regulation of stomatal opening	BP	1.73E-06	4.40E-09	26
GO:0010030	positive regulation of seed germination	BP	1.87E-06	4.95E-09	26
GO:0006469	negative regulation of protein kinase activity	BP	2.65E-06	7.94E-09	27
GO:0008126	acetylesterase activity	MF	9.36E-06	2.99E-08	12
GO:0050285	sinapine esterase activity	MF	1.09E-05	3.60E-08	13
GO:0004722	protein serine/threonine	MF	1.35E-05	4.66E-08	29

	phosphatase activity				
GO:0003144	embryonic heart tube formation	BP	1.94E-05	7.54E-08	5
GO:1901202	negative regulation of extracellular matrix assembly	BP	1.94E-05	7.54E-08	5
GO:0007505	adult fat body development	BP	1.94E-05	7.54E-08	5
GO:0007561	imaginal disc eversion	BP	1.94E-05	7.54E-08	5
GO:0034769	basement membrane disassembly	BP	1.94E-05	7.54E-08	5
GO:0097156	fasciculation of motor neuron axon	BP	1.94E-05	7.54E-08	5
GO:0035202	tracheal pit formation in open tracheal system	BP	1.94E-05	7.54E-08	5
GO:0103115	protoheme IX ABC transporter activity	MF	5.33E-05	2.15E-07	6
GO:0030247	polysaccharide binding	MF	7.58E-05	3.22E-07	34

Supplementary Table 12. Summary of the gene classification of all genes and genes in the expanded gene families in *C. acuminata* genome.

	**All genes**	**Percentage (%)**	**Genes in the expanded gene families**	**Percentage (%)**
Total	27,940	100	9,249	100
Singleton	3,655	13.08	32	0.34
Dispersed	4,660	16.68	810	8.76
Proximal	844	3.02	253	2.74
Tandem	1,768	6.33	695	7.51
WGD or segmental	17,013	60.89	7,458	80.64

Supplementary Table 13. GO enrichment analysis of expanded gene families in the *C. acuminata* genome. Significance was tested by two tailed Fisher's exact test method.

**GO ID**	**GO name**	**GO category**	**FDR**	***p*-value**	**Number of genes**
GO:0010200	response to chitin	BP	1.67E-24	9.61E-27	570
GO:0009911	positive regulation of flower development	BP	5.18E-21	3.72E-23	248
GO:0042803	protein homodimerization activity	MF	1.33E-18	1.16E-20	859
GO:0010268	brassinosteroid homeostasis	BP	2.10E-15	2.23E-17	128
GO:0080092	regulation of pollen tube growth	BP	3.56E-15	3.84E-17	138
GO:0005509	calcium ion binding	MF	2.54E-14	3.10E-16	364
GO:0033466	trans-zeatin biosynthetic process	BP	8.35E-14	1.08E-15	39
GO:0016185	synaptic vesicle budding from presynaptic endocytic zone membrane	BP	1.46E-12	2.15E-14	44
GO:0005388	calcium-transporting ATPase activity	MF	1.82E-12	2.72E-14	40
GO:0050616	secologanin synthase activity	MF	2.71E-11	4.60E-13	35
GO:0008395	steroid hydroxylase activity	MF	4.84E-11	8.53E-13	56
GO:0009846	pollen germination	BP	8.08E-11	1.47E-12	155
GO:0042538	hyperosmotic salinity response	BP	8.12E-11	1.48E-12	230
GO:0046777	protein autophosphorylation	BP	1.21E-10	2.27E-12	591
GO:0016760	cellulose synthase (UDP-forming) activity	MF	1.31E-10	2.47E-12	42
GO:1902382	11-oxo-beta-amyrin catabolic process	BP	1.50E-10	2.94E-12	35
GO:1902386	glycyrrhetinate biosynthetic process	BP	1.50E-10	2.94E-12	35
GO:0102375	11-oxo-beta-amyrin 30-oxidase activity	MF	1.50E-10	2.94E-12	35
GO:0010400	rhamnogalacturonan I side chain metabolic process	BP	2.87E-10	5.86E-12	41
GO:0008017	microtubule binding	MF	3.46E-10	7.13E-12	241

GO:0048768	root hair cell tip growth	BP	3.87E-10	8.03E-12	65
GO:0010305	leaf vascular tissue pattern formation	BP	7.23E-10	1.56E-11	217
GO:0009877	nodulation	BP	8.09E-10	1.76E-11	143
GO:0050321	tau-protein kinase activity	MF	8.85E-10	1.93E-11	29
GO:0015386	potassium:proton antiporter activity	MF	1.55E-09	3.50E-11	46
GO:1903288	positive regulation of potassium ion import	BP	2.21E-09	5.09E-11	28
GO:0036377	arbuscular mycorrhizal association	BP	4.85E-09	1.17E-10	60
GO:0003785	actin monomer binding	MF	7.23E-09	1.78E-10	37
GO:0004565	beta-galactosidase activity	MF	1.07E-08	2.68E-10	52
GO:0010483	pollen tube reception	BP	1.45E-08	3.68E-10	48

Supplementary Table 14. GO enrichment analysis of tandem repeat genes in the *C. acuminata* genome. Significance was tested by two tailed Fisher's exact test method.

GO ID	GO name	GO category	FDR	*p*-value	Number of genes
GO:0042432	indole biosynthetic process	BP	1.03E-47	8.87E-51	96
GO:0080043	quercetin 3-*O*-glucosyltransferase activity	MF	6.24E-39	1.10E-41	88
GO:0080044	quercetin 7-*O*-glucosyltransferase activity	MF	6.24E-39	1.10E-41	88
GO:0009626	plant-type hypersensitive response	BP	1.44E-34	2.92E-37	197
GO:0050403	trans-zeatin *O*-beta-D-glucosyltransferase activity	MF	3.00E-33	7.05E-36	58
GO:0050502	cis-zeatin *O*-beta-D-glucosyltransferase activity	MF	7.32E-33	1.79E-35	58
GO:0009718	anthocyanin-containing compound biosynthetic process	BP	6.10E-28	1.82E-30	136
GO:1900994	(-)-secologanin biosynthetic process	BP	2.88E-27	8.87E-30	42
GO:0050057	linamarin synthase activity	MF	1.95E-25	6.44E-28	38
GO:0080167	response to karrikin	BP	4.69E-25	1.59E-27	199
GO:0009816	defense response to bacterium, incompatible interaction	BP	2.29E-24	8.39E-27	139
GO:0102425	myricetin 3-*O*-glucosyltransferase activity	MF	4.26E-24	1.62E-26	48
GO:0102360	daphnetin 3-*O*-glucosyltransferase activity	MF	4.26E-24	1.62E-26	48
GO:0047893	flavonol 3-*O*-glucosyltransferase activity	MF	4.26E-24	1.62E-26	48
GO:0020037	heme binding	MF	8.85E-22	3.76E-24	125
GO:0005506	iron ion binding	MF	2.16E-21	9.28E-24	130
GO:0009817	defense response to fungus, incompatible interaction	BP	1.32E-20	6.01E-23	93
GO:0052640	salicylic acid glucosyltransferase (glucoside-forming) activity	MF	3.60E-20	1.68E-22	37
GO:0009805	coumarin biosynthetic process	BP	5.84E-20	2.82E-22	78
GO:0050506	vomilenine glucosyltransferase activity	MF	5.84E-20	2.82E-22	29

GO:0050247	raucaffricine beta-glucosidase activity	MF	5.84E-20	2.82E-22	29
GO:0033329	kaempferol *O*-glucoside metabolic process	BP	1.00E-19	5.11E-22	30
GO:0033302	quercetin *O*-glucoside metabolic process	BP	1.00E-19	5.11E-22	30
GO:0033956	beta-apiosyl-beta-glucosidase activity	MF	1.29E-18	6.73E-21	31
GO:0102970	7-deoxyloganetic acid glucosyltransferase activity	MF	1.50E-18	7.85E-21	26
GO:0019762	glucosinolate catabolic process	BP	6.75E-18	3.90E-20	34
GO:0010132	dhurrin biosynthetic process	BP	6.75E-18	3.83E-20	29
GO:0033907	beta-D-fucosidase activity	MF	8.27E-18	4.93E-20	31
GO:0047701	beta-L-arabinosidase activity	MF	8.27E-18	4.93E-20	31
GO:0080083	beta-gentiobiose beta-glucosidase activity	MF	8.27E-18	4.93E-20	31

Supplementary Table 15. Benchmarking Universal Single-Copy Orthologs (BUSCO) analysis about caa_assembly_v_10072011 and *C. acuminata* V3.0.

	**Total BUSCO groups searched**	**Complete single-copy BUSCOs**	**Complete duplicated BUSCOs**	**Fragmented BUSCOs**	**Missing BUSCOs**	**Complete BUSCOs Percentage (%)**
caa_assembly_v_ 10072011	1440	554	686	89	111	86.1
C. acuminata V3.0	1440	1270	97	21	52	94.9

Supplementary Table 16. Accession numbers and sequence identities of *C. acuminata* candidate genes involved in the biosynthesis of Camptothecin.

Description	Abbreviation	Query	Top-hit *C. acuminata* gene ID	Identity (%)	Coverage (%)
Anthranilate synthase	ASA	NP_196192.1	CacGene15948	73.01	98.48
Anthranilate phosphoribosyltransferase	APT	NP_001185362.1	CacGene17722	69.63	100
Phosphoribosylanthranilate isomerase	PAI	NP_001184931.1	CacGene19966	68.57	73.09
Indole-3-glycerol phosphate synthase	IGPS	NP_178521.1	CacGene19211	67.66	99.50
Tryptophan synthase	TS-alpha chain	NP_567004.1	CacGene24069	69.84	96.79
	TS-beta chain	NP_194437.1	CacGene25673	80.34	99.58
Tryptophan decarboxylase	TDC	AAA33109.1	CacGene21142（TDC1）<br>CacGene13733（TDC2）	69.15<br>67.35	96.20<br>97.60
1-Deoxy-D-xylulose-5-phosphate synthase	DXS	ABI35993.1	CacGene07953	77.60	96.14
1-Deoxy-D-xylulose-5-phosphate reductoisomerase	DXR	AAF65154.1	CacGene14522	88.82	98.34
4-Diphosphocytidyl-2-C-methyl-D-erythritol synthase	CMS	ACI16377	CacGene08828	77.82	86.75
4-Diphosphocytidyl-2-C-methyl-D-erythritol kinase	CMK	ABI35992.1	CacGene00537	83.28	73.49
2C-Methyl-D-erythritol 2,4-cyclodiphosphate synthase	MCS	AAF65155.1	CacGene21954	73.77	97.92
1-Hydroxy-2-methyl-2-butenyl-4-diphosphate synthase	HDS	AAO24774.1	CacGene11201	89.19	98.40

1-Hydroxy-2-methyl-butenyl 4-diphosphate reductase	HDR	ABI30631.1	CacGene22107	83.77	98.30
Isopentenyl diphosphate isomerase	IDI	ABW98669.1	CacGene09399	80.67	84.85
geranyl pyrophosphate synthase	GPS	ACC77966.1	CacGene21163	77.14	98.59
Geraniol synthase	GS	AFD64744	CacGene15279	65.16	92.14
geraniol 8-hydroxylase	G8H	CAC80883.1	CacGene19461	60.40	98.00
8-hydroxygeraniol oxidoreductase	8HO	AHK60836.1	CacGene09426	71.69	98.18
CYC1(Iridoid synthase)	CYC1(IS)	AFW98981.1	CacGene01310	65.90	98.48
7-Deoxyloganetic acid synthase	7-DLS	AHX24370.1	CacGene21929	78.44	95.61
7-Deoxyloganetic acid-*O*-glucosyltransferase	7-DLGT	AHK60835.1	CacGene15981	77.80	94.89
7-Deoxyloganic acid 7-hydroxylase	7-DLH	AGX93062.1	CacGene13171	69.58	94.71
loganic acid methyltransferase	LAMT	ABW38009.1	CacGene05741	53.91	93.37
secologanin synthase	SLS	AAA33106.1	CacGene10833	63.36	98.31
Strictosidine synthase	STS	P18417.2	CacGene06084	41.13	78.49

Supplementary Table 17. Gene copy number of enzymes involved in the biosynthesis of Camptothecin in 8 selected plant species by ortholog groups.

EC number	Enzyme name	Vvi	Cca	Ath	Cac	Dca	Cro	Lsa	Rde	Ortholog group
4.1.3.27	ASA	2	2	3	3	3	2	2	4	OCG1052
2.4.2.18	APT	1	1	1	1	1	1	1	1	OCG8977
5.3.1.24	PAI	1	1	3	1	1	1	1	1	OCG4374
4.1.1.48	IGPS	1	1	2	1	1	2	2	1	OCG4258
4.2.1.20	TS-alpha chain	1	1	2	1	1	1	1	1	OCG7519
	TS-beta chain	2	2	3	2	1	2	2	5	OCG1289
4.1.1.28	TDC	3	7	2	6	8	3	5	1	OCG439*
2.2.1.7	DXS	12	6	10	14	13	8	10	6	OCG113*
1.1.1.267	DXR	1	2	1	2	1	1	2	1	OCG3911*
2.7.7.60	CMS	1	1	1	1	2	1	1	1	OCG6631
2.7.1.148	CMK	1	1	1	1	1	1	1	1	OCG9196
4.6.1.12	MCS	1	1	1	1	1	1	1	1	OCG8917
1.17.7.1<br>1.17.7.3	HDS	1	1	1	2	2	1	1	1	OCG4846*
1.17.7.4	HDR	1	1	1	1	3	1	2	3	OCG2846
5.3.3.2	IDI	1	1	2	1	1	1	1	1	OCG5899
2.5.1.1	GPS	1	1	1	1	1	1	1	2	OCG7218
3.1.7.11	GS	2	2	0	1	0	1	1	1	OCG8068
1.14.13.152	G8H	10	6	7	3	5	4	22	11	OCG149
1.1.1.324	8HO	3	1	0	2	1	1	1	2	OCG3957*
	CYC1(IS)	1	3	1	5	4	4	2	3	OCG903*
	7-DLS	6	3	1	5	17	4	2	5	OCG311
	7-DLGT	4	9	0	6	0	7	6	6	OCG377*
	7-DLH/SLS	20	35	8	18	4	16	21	8	OCG55*
	LAMT	0	0	4	1	0	1	0	4	OCG4731

*Ontholog groups which are expanded in *C. acuminata.* Source data are provided as a Source data file.

Supplementary Table 18. Genes in gray60 modules with weight over 0.25 and their function annotation.

Gene ID	Degree (Hub-level)	Function annotation by SWISS-PROT
CacGene27626	83	SABP2_TOBAC Salicylic acid-binding 2 OS=Nicotiana tabacum GN=SABP2 PE=1 SV=1
CacGene21929	81	C76A2_SOLME Cytochrome P450 76A2 OS=Solanum melongena GN=CYP76A2 PE=2 SV=1
CacGene01309	80	5BPOR_DIGLA 3-oxo-Delta(4,5)-steroid 5-beta-reductase OS=Digitalis lanata PE=1 SV=1
CacGene10416	80	BH025_ARATH Transcription factor bHLH25 OS=Arabidopsis thaliana GN=BHLH25 PE=2 SV=2
CacGene01310	79	IRIS_CATRO Iridoid synthase OS=Catharanthus roseus PE=1 SV=1
CacGene02631	77	IN22_MAIZE IN2-2 OS=Zea mays GN=IN2-2 PE=2 SV=1
CacGene07617	77	LSH3_ARATH LIGHT-DEPENDENT SHORT HYPOCOTYLS 3 OS=Arabidopsis thaliana GN=LSH3 PE=1 SV=1
CacGene15336	77	---NA---
CacGene02632	76	AKR2_ORYSJ Probable aldo-keto reductase 2 OS=Oryza sativa japonica GN=Os04g0338000 PE=2 SV=2
CacGene05419	76	C76A2_SOLME Cytochrome P450 76A2 OS=Solanum melongena GN=CYP76A2 PE=2 SV=1
CacGene15279	76	TPS11_RICCO Probable terpene synthase 11 OS=Ricinus communis GN=TPS11 PE=3 SV=1
CacGene10059	74	PGLR4_ARATH Polygalacturonase At1g48100 OS=Arabidopsis thaliana GN=At1g48100 PE=2 SV=1
CacGene09376	73	MYBC_MAIZE Anthocyanin regulatory C1 OS=Zea mays GN=C1 PE=2 SV=1
CacGene07206	72	---NA---
CacGene09135	72	TPS32_SOLLC Viridiflorene synthase OS=Solanum lycopersicum GN=TPS32 PE=1 SV=1
CacGene18745	71	TRXH2_ARATH Thioredoxin H2 OS=Arabidopsis thaliana GN=TRX2 PE=2 SV=2
CacGene16081	68	LSH10_ARATH LIGHT-DEPENDENT SHORT HYPOCOTYLS 10 OS=Arabidopsis thaliana GN=LSH10 PE=1 SV=1
CacGene16906	68	ZIP1_ARATH Zinc transporter 1 OS=Arabidopsis thaliana GN=ZIP1 PE=2 SV=1
CacGene00105	66	EXB15_ORYSJ Expansin-B15 OS=Oryza sativa japonica GN=EXPB15 PE=3 SV=2
CacGene00948	66	ATESY_VITVI (-)-alpha-terpineol synthase OS=Vitis vinifera PE=1 SV=1
CacGene12065	64	DREB3_ARATH Dehydration-responsive element-binding 3 OS=Arabidopsis thaliana GN=DREB3 PE=2 SV=1

CacGene22177	64	---NA---
CacGene13171	63	C7A29_PANGI Cytochrome P450 CYP72A219 OS=Panax ginseng PE=2 SV=1
CacGene14429	63	BH096_ARATH Transcription factor bHLH96 OS=Arabidopsis thaliana GN=BHLH96 PE=2 SV=1
CacGene09428	62	ADHL1_ARATH Alcohol dehydrogenase-like 1 OS=Arabidopsis thaliana GN=At1g22430 PE=2 SV=1
CacGene02917	61	HFA6B_ARATH Heat stress transcription factor A-6b OS=Arabidopsis thaliana GN=HSFA6b PE=2 SV=1
CacGene04626	61	LBD1_ARATH LOB domain-containing 1 OS=Arabidopsis thaliana GN=LBD1 PE=2 SV=1
CacGene26584	61	LSH6_ARATH LIGHT-DEPENDENT SHORT HYPOCOTYLS 6 OS=Arabidopsis thaliana GN=LSH6 PE=1 SV=1
CacGene14783	60	IQD14_ARATH IQ-DOMAIN 14 OS=Arabidopsis thaliana GN=IQD14 PE=1 SV=1
CacGene05598	59	DXR_MENPI 1-deoxy-D-xylulose 5-phosphate chloroplastic OS=Mentha piperita GN=DXR PE=2 SV=2
CacGene14542	59	AKT1_ARATH Potassium channel AKT1 OS=Arabidopsis thaliana GN=AKT1 PE=1 SV=2
CacGene10972	58	HIP35_ARATH Heavy metal-associated isoprenylated plant 35 OS=Arabidopsis thaliana GN=HIPP35 PE=2 SV=1
CacGene23928	58	LSH10_ARATH LIGHT-DEPENDENT SHORT HYPOCOTYLS 10 OS=Arabidopsis thaliana GN=LSH10 PE=1 SV=1
CacGene06196	57	INV1_ARATH Beta- insoluble isoenzyme CWINV1 OS=Arabidopsis thaliana GN=CWINV1 PE=1 SV=1
CacGene22551	56	PIRL_SOLLC Pirin OS=Solanum lycopersicum PE=2 SV=1
CacGene15981	54	UGT8_CATRO 7-deoxyloganetic acid glucosyltransferase OS=Catharanthus roseus GN=UGT709C2 PE=1 SV=1
CacGene25245	54	CYCLD_ANTMA Transcription factor CYCLOIDEA OS=Antirrhinum majus GN=CYC PE=1 SV=1
CacGene02914	53	EPFL4_ARATH EPIDERMAL PATTERNING FACTOR 4 OS=Arabidopsis thaliana GN=EPFL4 PE=1 SV=1
CacGene02721	52	TRM7_DICDI tRNA (cytidine(32) guanosine(34)-2 -O)-methyltransferase OS=Dictyostelium discoideum GN=fsjA PE=3 SV=1
CacGene02620	51	BOR2_ARATH Probable boron transporter 2 OS=Arabidopsis thaliana GN=BOR2 PE=2 SV=1
CacGene13909	51	HIP26_ARATH Heavy metal-associated isoprenylated plant 26 OS=Arabidopsis thaliana GN=HIPP26 PE=1 SV=1
CacGene18693	51	RSS3_ORYSJ RICE SALT SENSITIVE 3 OS=Oryza sativa japonica GN=RSS3 PE=1 SV=1
CacGene19215	51	AB1K7_ARATH ACTIVITY OF BC1 COMPLEX KINASE

		chloroplastic OS=Arabidopsis thaliana GN=ABC1K7 PE=2 SV=1
CacGene20894	49	ZWIP3_ARATH Zinc finger WIP3 OS=Arabidopsis thaliana GN=WIP3 PE=2 SV=1
CacGene23366	49	AHP4_ARATH Histidine-containing phosphotransfer 4 OS=Arabidopsis thaliana GN=AHP4 PE=1 SV=2
CacGene01655	48	BGL47_ARATH Beta-glucosidase 47 OS=Arabidopsis thaliana GN=BGLU47 PE=3 SV=2
CacGene21020	48	BRG1_ARATH BOI-related E3 ubiquitin- ligase 1 OS=Arabidopsis thaliana GN=BRG1 PE=1 SV=1
CacGene18277	47	RAY1_ARATH Beta-arabinofuranosyltransferase RAY1 OS=Arabidopsis thaliana GN=RAY1 PE=2 SV=1
CacGene19123	44	AZG2_ARATH Adenine guanine permease AZG2 OS=Arabidopsis thaliana GN=AZG2 PE=2 SV=1
CacGene17278	43	F3PH_ARATH Flavonoid 3 -monooxygenase OS=Arabidopsis thaliana GN=CYP75B1 PE=1 SV=1
CacGene23476	42	SBT56_ARATH Subtilisin-like protease OS=Arabidopsis thaliana GN= PE=2 SV=1
CacGene10013	41	FTRC1_SPIOL Ferredoxin-thioredoxin reductase catalytic chloroplastic OS=Spinacia oleracea GN=FTRC PE=1 SV=2
CacGene06978	39	NUD20_ARATH Nudix hydrolase chloroplastic OS=Arabidopsis thaliana GN=NUDT20 PE=2 SV=1
CacGene20462	39	ZWIP2_ARATH Zinc finger WIP2 OS=Arabidopsis thaliana GN=WIP2 PE=1 SV=1
CacGene10425	38	BH093_ARATH Transcription factor bHLH93 OS=Arabidopsis thaliana GN=BHLH93 PE=1 SV=1
CacGene26945	32	YUC6_ARATH Indole-3-pyruvate monooxygenase YUCCA6 OS=Arabidopsis thaliana GN=YUC6 PE=1 SV=1
CacGene07629	31	PHT17_ARATH Probable inorganic phosphate transporter 1-7 OS=Arabidopsis thaliana GN=PHT1-7 PE=2 SV=2
CacGene12748	31	SUT13_ARATH Sulfate transporter OS=Arabidopsis thaliana GN=SULTR1 3 PE=2 SV=1
CacGene03019	29	METK5_VITVI S-adenosylmethionine synthase 5 OS=Vitis vinifera GN=METK5 PE=3 SV=1
CacGene15528	29	CHX20_ARATH Cation H(+) antiporter 20 OS=Arabidopsis thaliana GN=CHX20 PE=2 SV=1
CacGene04171	28	CCD11_ARATH Cyclin-D1-1 OS=Arabidopsis thaliana GN=CYCD1-1 PE=1 SV=3
CacGene09430	27	ADHL3_ARATH Alcohol dehydrogenase-like 3 OS=Arabidopsis thaliana GN=At1g32780 PE=2 SV=1
CacGene03548	25	---NA---
CacGene04103	25	PLRX3_ARATH Pollen-specific leucine-rich repeat extensin 3 OS=Arabidopsis thaliana GN=PEX3 PE=2 SV=1

CacGene08463	24	IQM2_ARATH IQ domain-containing IQM2 OS=Arabidopsis thaliana GN=IQM2 PE=2 SV=1
CacGene15721	23	DEF02_ARATH Defensin 2 OS=Arabidopsis thaliana GN= PE=2 SV=1
CacGene25108	22	GDL6_ARATH GDSL esterase lipase At1g28570 OS=Arabidopsis thaliana GN=At1g28570 PE=3 SV=1
CacGene24459	20	CYB5_TOBAC Cytochrome b5 OS=Nicotiana tabacum PE=2 SV=1
CacGene26195	20	LAC14_ARATH Laccase-14 OS=Arabidopsis thaliana GN=LAC14 PE=2 SV=1
CacGene16153	19	GIS3_ARATH Zinc finger GIS3 OS=Arabidopsis thaliana GN=GIS3 PE=2 SV=1
CacGene22926	19	Y5258_ARATH Stress-response A B barrel domain-containing At5g22580 OS=Arabidopsis thaliana GN=At5g22580 PE=1 SV=1
CacGene19847	18	---NA---
CacGene06691	17	EXPB2_ARATH expansin-B2 OS=Arabidopsis thaliana GN=EXPB2 PE=3 SV=2
CacGene22059	16	GLGL1_SOLTU Glucose-1-phosphate adenylyltransferase large subunit 1 (Fragment) OS=Solanum tuberosum GN=AGPS1 PE=2 SV=1
CacGene17921	13	KCO1_ARATH Two-pore potassium channel 1 OS=Arabidopsis thaliana GN=TPK1 PE=1 SV=2
CacGene26870	12	---NA---
CacGene05420	10	C76A2_SOLME Cytochrome P450 76A2 OS=Solanum melongena GN=CYP76A2 PE=2 SV=1
CacGene23478	10	SBT55_ARATH Subtilisin-like protease OS=Arabidopsis thaliana GN= PE=3 SV=2
CacGene08793	9	APA1_ARATH Aspartic ase A1 OS=Arabidopsis thaliana GN=APA1 PE=1 SV=1
CacGene05289	8	RAP27_ARATH Ethylene-responsive transcription factor RAP2-7 OS=Arabidopsis thaliana GN=RAP2-7 PE=2 SV=2
CacGene06910	8	RDR3_ARATH Probable RNA-dependent RNA polymerase 3 OS=Arabidopsis thaliana GN=RDR3 PE=3 SV=2
CacGene20120	6	DIR23_ARATH Dirigent 23 OS=Arabidopsis thaliana GN=DIR23 PE=2 SV=1
CacGene08470	5	---NA---
CacGene05421	4	C76A2_SOLME Cytochrome P450 76A2 OS=Solanum melongena GN=CYP76A2 PE=2 SV=1
CacGene08325	4	SRS5_ARATH SHI RELATED SEQUENCE 5 OS=Arabidopsis thaliana GN=SRS5 PE=2 SV=1
CacGene04487	2	---NA---

Supplementary Table 19. Summary of RNA sequencing data.

SRA run number	Tissue	Read type	Number of reads	Total data (Gb)	Read length (bp)	Mapping rate (%)	Use
SRR173032	Whole seedlings	Illumina paired-end	20,222,772	2.22	2 x 55	72.52	expression level calculation
SRR173033	Young leaf	Illumina paired-end	44,155,958	6.62	2 x 75	67.25	expression level calculation
SRR173034	Mature leaf	Illumina paired-end	45,437,386	6.82	2 x 75	92.09	transcriptome assembly, expression level calculation
SRR173035	Immature bark	Illumina paired-end	42,272,958	6.34	2 x 75	93.49	transcriptome assembly, expression level calculation
SRR173036	Entire root	Illumina paired-end	44,473,260	6.67	2 x 75	91.50	transcriptome assembly, expression level calculation
SRR173042	Callus	Illumina single-end	25,812,941	0.90	35	93.30	expression level calculation
SRR173043	Root culture	Illumina single-end	24,961,098	0.87	35	92.70	expression level calculation
SRR173044	Young flower	Illumina paired-end	36,383,996	5.46	2 x 75	90.00	transcriptome assembly, expression level calculation
SRR173045	Immature fruit	Illumina paired-end	42,604,968	6.39	2 x 75	87.66	transcriptome assembly, expression level calculation
SRR173046	Mature fruit	Illumina paired-end	25,101,356	3.77	2 x 75	85.90	transcriptome assembly, expression level calculation
SRR173047	Trichomes	Illumina paired-end	40,748,840	6.11	2 x 75	72.02	expression level calculation
SRR173048	Advance roots	Illumina paired-end	44,469,609	6.67	2 x 75	90.63	expression level calculation
SRR173052	Cotyledons	Illumina paired-end	37,086,312	5.56	2 x 75	92.35	transcriptome assembly, expression level calculation

SRR173053	Upper stem	Illumina paired-end	38,056,744	5.71	2 x 75	94.01	transcriptome assembly, expression level calculation
SRR173054	Lower stem	Illumina paired-end	36,342,362	5.45	2 x 75	96.09	transcriptome assembly, expression level calculation

Supplementary Table 20. Primers used for amplification of CrLAMT and CaLAMT.

Primer name	Sequence (5’ to 3’)
CrLAMT-KF415116_F	ATGGTTGCCACAATTGA
CrLAMT-KF415116_R	TTAATTTCCCTTGCGTTTCAAG
CaLAMT-CacGene05741_F	ATGGCTAATGAGGAGCCCA
CaLAMT-CacGene05741_R	TTAATCAGTCATTTTACGTTTAAG

Supplementary Table 21. Primers used for construct expression vector of CrLAMT, CaLAMT and CrLAMT mutates. For expression of recombinant proteins in yeast using pESC-His vector. Underlined sequences mean restriction sites, and bold sequences mean mutations from CrLAMT (KF415116).

Primer name	Sequence (5' to 3')
pESC-CrLAMT-KF415116_F	CTATAGGGCCCGGGC GTCGAC<br>ATGGTTGCCACAATTGA
pESC-CrLAMT-KF415116_R	GCGGTACCAAGCTTA CTCGAG<br>TTAATGATGGTGATGGTGATG<br>ATTTCCCTTGCGTTTCAAG
pESC-CaLAMT-CacGene05741_F	CTATAGGGCCCGGGCGTCGAC<br>ATGGCTAATGAGGAGCCCA
pESC-CaLAMT-CacGene05741_R	GCGGTACCAAGCTTA CTCGAG<br>TTAATGATGGTGATGGTGATG<br>ATCAGTCATTTTACGTTTAAG
pESC-CrLAMT-KF415116-G240C_F	CTTCACTTCCTTTTGGGAACT
pESC-CrLAMT-KF415116-G240C_R	AGTTCCCAAAAGGAAGTGAAGCAAACCAGC<br>AC**A**AGTCCTTG
pESC-CrLAMT-KF415116-A241T_F	CTTCACTTCCTTTTGGGAACT
pESC-CrLAMT-KF415116-A241T_R	AGTTCCCAAAAGGAAGTGAAGCAAACCAG**T**<br>ACCAGTCCT
pESC-CrLAMT-KF415116-G242S_F	TCACTTCCTTTTGGGAACTTCCT
pESC-CrLAMT-KF415116-G242S_R	AGGAAGTTCCCAAAAGGAAGTGAAGCAAAC<br>**T**AGCACCAGTCCT
pESC-CrLAMT-KF415116-L243M_F	CTTCCTTTTGGGAACTTCCT
pESC-CrLAMT-KF415116-L243M_R	AGGAAGTTCCCAAAAGGAAGTGAAGCA**T**AC<br>CAGCACCAGTCCT

pESC-CrLAMT-KF415116-H245F_F	TTGGGAACTTCCTTGATGGA
pESC-CrLAMT-KF415116-H245F_R	TCCATCAAGGAAGTTCCCAAAAGGAA**AAA**A AGCAAACCAGCACCAG
