# Supplementary material for: A chromosome-level Camptotheca acuminata genome assembly provides insights into the evolutionary origin of camptothecin biosynthesis
Source: Nat Commun. 2021 Jun 10;12:3531. doi: 10.1038/s41467-021-23872-9 (PMC8192753; doi:10.1038/s41467-021-23872-9)
Supplement: Supplementary file 4 — Description of Additional Supplementary Files [file 41467_2021_23872_MOESM4_ESM.pdf]

**Description of additional supplementary files**

Title: Supplementary Data 1
Description: Protein-coding genes may have wrong structure annotation in *Cac* genome assembly v2.4 compare with *C. acuminata* V3.0.

Title: Supplementary Data 2
Description: Expression abundance matrix (fragments per kb exon model per million mapped reads) from 15 tissues of *C. acuminata*.

Title: Supplementary Data 3
Description: Differential expressed genes and their expression abundance matrix (fragments per kb exon model per million mapped reads) from 15 tissues of *C. acuminata*.

Title: Supplementary Data 4
Description: Genes classified into 30 modules in different colors by WGCNA.

Title: Supplementary Data 5
Description: Selection analysis of LAMT using the branch site model (BSM). Significance was tested by two tailed likelihood ratio test (LRT) method.

Title: Supplementary Data 6
Description: Selection analysis of LAMT using the branch model (BM). Significance was tested by two tailed likelihood ratio test (LRT) method.

Title: Supplementary Data 7
Description: Summary of relative activity of the LAMTs.

Title: Supplementary Data 8
Description: Selection analysis of SLS/SLAS using the branch site model (BSM). Significance was tested by two tailed likelihood ratio test (LRT) method.
